# Cheminformatics-aided discovery of small-molecule Protein-Protein Interaction (PPI) dual inhibitors of Tumor Necrosis Factor (TNF) and Receptor Activator of NF-κB Ligand (RANKL)

**DOI:** 10.1371/journal.pcbi.1005372

**Published:** 2017-04-20

**Authors:** Georgia Melagraki, Evangelos Ntougkos, Vagelis Rinotas, Christos Papaneophytou, Georgios Leonis, Thomas Mavromoustakos, George Kontopidis, Eleni Douni, Antreas Afantitis, George Kollias

**Affiliations:** 1 Division of Immunology, Biomedical Sciences Research Center 'Alexander Fleming', Vari, Greece; 2 NovaMechanics Ltd, Nicosia, Cyprus; 3 Laboratory of Genetics, Department of Biotechnology, Agricultural University of Athens, Athens, Greece; 4 Veterinary School, University of Thessaly, Karditsa, Greece; 5 Institute for Research and Technology Thessaly (IRETETH), Volos, Greece; 6 Department of Life and Health Sciences, School of Sciences and Engineering, University of Nicosia, Nicosia, Cyprus; 7 Laboratory of Organic Chemistry, Department of Chemistry, National and Kapodistrian University of Athens, Athens, Greece; 8 Department of Experimental Physiology, Medical School, National and Kapodistrian University of Athens, Athens, Greece; Baltimore, UNITED STATES

## Abstract

We present an *in silico* drug discovery pipeline developed and applied for the identification and virtual screening of small-molecule Protein-Protein Interaction (PPI) compounds that act as dual inhibitors of TNF and RANKL through the trimerization interface. The cheminformatics part of the pipeline was developed by combining structure–based with ligand–based modeling using the largest available set of known TNF inhibitors in the literature (2481 small molecules). To facilitate virtual screening, the consensus predictive model was made freely available at: http://enalos.insilicotox.com/TNFPubChem/. We thus generated a priority list of nine small molecules as candidates for direct TNF function inhibition. *In vitro* evaluation of these compounds led to the selection of two small molecules that act as potent direct inhibitors of TNF function, with IC_50_ values comparable to those of a previously-described direct inhibitor (SPD304), but with significantly reduced toxicity. These molecules were also identified as RANKL inhibitors and validated *in vitro* with respect to this second functionality. Direct binding of the two compounds was confirmed both for TNF and RANKL, as well as their ability to inhibit the biologically-active trimer forms. Molecular dynamics calculations were also carried out for the two small molecules in each protein to offer additional insight into the interactions that govern TNF and RANKL complex formation. To our knowledge, these compounds, namely T8 and T23, constitute the second and third published examples of dual small-molecule direct function inhibitors of TNF and RANKL, and could serve as lead compounds for the development of novel treatments for inflammatory and autoimmune diseases.

## Author summary

Developing drugs that disrupt protein-protein interactions (PPIs) is a difficult task in pharmaceutical research. The interaction between protein Tumor Necrosis Factor (TNF) and its receptors is implicated in several physiological functions and diseases, such as rheumatoid and psoriatic arthritis, Crohn’s disease, and multiple sclerosis. Despite their potency, current medications that block the interaction between TNF and its receptors are also associated with many adverse functions. Here, we employ comprehensive computational and experimental methods to discover novel small molecules that are direct inhibitors of TNF function. Functionality for RANKL, a second, clinically-relevant member of the TNF protein family, was also examined. Using a combination of an *in silico* drug discovery pipeline, which includes structure- and ligand-based modeling, and *in vitro* experiments, we identified compounds T8 and T23 as dual inhibitors of TNF and RANKL. These compounds present low toxicity and may be further optimized in drug design targeting TNF and RANKL to develop improved treatments for a range of inflammatory and autoimmune diseases.

## Introduction

Tumor Necrosis Factor (TNF) is a pro-inflammatory cytokine[1] that is associated with a variety of important physiological processes and pathological conditions, including rheumatoid arthritis (RA), psoriatic arthritis, Crohn’s disease, and multiple sclerosis.[2,3] To control the adverse functions of TNF, efforts have focused on blocking TNF binding to its receptors.[4] TNF is generated as a transmembrane protein (tmTNF), which is proteolytically cleaved by tumor necrosis factor-α-converting enzyme (TACE) to its soluble form (sTNF).[5] sTNF and tmTNF bind to two different receptors, TNFR1 (TNF receptor type 1) and TNFR2 (TNF receptor type 2) with differential capacities, exerting differential functions.[6,7] While TNFR1 is expressed on most cell types, TNFR2 is expressed mainly on immune cells[6] and its complete activation requires the presence of tmTNF.[8] It has been demonstrated that tmTNF and sTNF differ in their physiological functions[9,10] and inhibitors that distinctively target them may result in differential effects.[11] Early studies in our lab provided *in vivo* evidence that deregulated TNF production is causal to the development of chronic polyarthritis in a transgenic animal model and that anti-TNF treatment is efficacious for treating the modeled disease.[12] These studies were instrumental in mobilizing the interest of the anti-TNF industry, which led to the first successful clinical trials performed initially for RA[13] and then for other chronic inflammatory diseases, such as Crohn’s disease, psoriasis, psoriatic arthritis, juvenile idiopathic arthritis, spondylarthritis and Behçet's disease.[14] Thus far, four synthetic antibodies have been approved by the FDA as effective TNF inhibitors, namely infliximab, adalimumab, certolizumab pegol[15] and golimumab[16] as well as the Fc-p75 receptor etanercept.[17] However, their discovery did not limit the ever-increasing research efforts towards the development of new anti-TNF drugs, mainly due to impediments, such as unwanted side effects (e.g. high risk of hepatitis B and tuberculosis), insufficient clinical response, the need for intravenous administration, and high cost. Drug development that leads to small-molecule inhibitors may overcome several of the above drawbacks by offering important advantages, such as oral administration, shorter half-lives with lower immunosuppression, easier manufacturing and reduced cost.[14]

**Fig 1:**
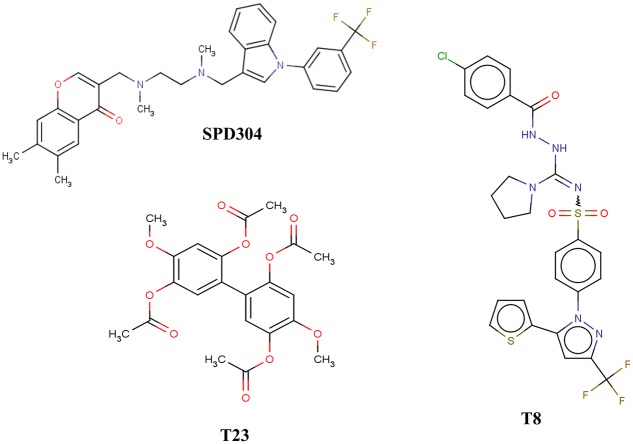
Chemical structures of compounds SPD304 (published inhibitor), and T8, T23 (the two most potent inhibitors identified by our pipeline).

The development of small-molecule inhibitors for protein-protein interactions (PPIs) is a nontrivial task in drug research.[18–21] Successful drug design requires the identification of compounds with low molecular weight, something extremely challenging, especially when attempting to block interactions between large molecules, such as proteins.[22] The successful recognition of small-molecule inhibitors is also hampered by the difficulty to identify potential “hot spots” as unique binding targets that are crucial for the disruption of biomolecular interactions.[23,24] Regarding TNF–TNF receptor interactions, the majority of small molecules proposed to date interact with TNF indirectly by down-regulating the expression of the protein; direct disruption of the interaction between TNF and its receptors has been reported only for a handful of small molecules.[22] These include suramin and its analogs,[25,26] as well as the indole-linked chromone SPD304 (Fig 1).[27] The biologically active state of TNF is a homotrimer; direct inhibition by SPD304 disrupts the binding of TNF dimer with the third subunit, thus preventing the formation of the active complex.[28,29] The binding site of the TNF dimer comprises mostly hydrophobic residues, and particularly glycine, leucine and tyrosine. Predominant interactions between the dimer and small molecules have been reported to be hydrophobic and shape-driven, since ligand structures need to be large enough to directly interact with both subunits and also to promote disassembly of the third subunit.[27,30]

Despite their promising effectiveness, the agents that directly disrupt the interaction between TNF and its receptor are also associated with adverse functions, such as low potency, poor selectivity and significant side effects (suramin),[31] as well as cell toxicity (SPD304).[32] Recently, additional small molecules have been proposed for direct TNF inhibition, namely two natural product-like small molecules, which are currently under hit-to-lead optimization studies,[30] two FDA–approved drugs (darifenacin and ezetimibe),[33] and a metal–based compound [iridium(III) biquinoline complex].[34]

Notwithstanding the aforementioned advancements, development of medications aiming at small-molecule inhibition of TNF with reduced toxic effects and high potency remains inadequate; this study has thus aimed at the identification of novel compounds that fit this desirable profile. We combined a variety of cheminformatics techniques,[35–37] such as structure-based virtual screening, ligand-based modeling and molecular dynamics (MD), with the experimental evaluation of selected compounds, to identify small-molecule candidates for TNF inhibition. 14,400 diverse drug-like compounds were initially virtually-screened[38] from the Maybridge HitFinder database[39] and were docked into the binding site of the TNF dimer (PDB ID: 2AZ5). The 30 compounds with the closest docking score and binding conformation to SPD304 were further filtered through a ligand-based predictive model based on the largest dataset available, containing 2481 known TNF inhibitors,[40] to afford 9 active compounds, which were subsequently tested *in vitro* to evaluate their potency against TNF. Our ligand-based model developed for the prediction of small-molecule TNF inhibition was made publicly available through the Enalos Cloud platform (http://enalos.insilicotox.com/TNFPubChem/). *In vitro* evaluation of functional inhibition and direct binding pointed to two compounds, namely T8 and T23, as the most potent TNF inhibitors (Fig 1).

RANKL (Receptor activator of nuclear factor kappa-B ligand), another member of the TNF superfamily, is the master mediator of osteoclast formation and bone resorption. Its specific inhibition with a monoclonal antibody (denosumab) effectively reduces the incidence of fractures in postmenopausal women[41] and emerges as the latest therapeutic achievement against osteoporosis. It has been demonstrated that SPD304 also binds to RANKL and inhibits RANKL-mediated osteoclastogenesis.[42] This motivated us to perform additional computations and biological assays for the T8 and T23 complexes with RANKL. *In vitro* proof of this second functionality thus established these two compounds as dual inhibitors[43] of TNF and RANKL. MD and free energy calculations for both structures and SPD304 in complex with TNF and RANKL were carried out to offer additional insight into the interactions that govern TNF and RANKL complex formation. Finally, direct and specific binding of the two compounds was confirmed both for TNF and RANKL, as well as their ability to inhibit the biologically-active trimer forms.

## Materials and methods

**Fig 2:**
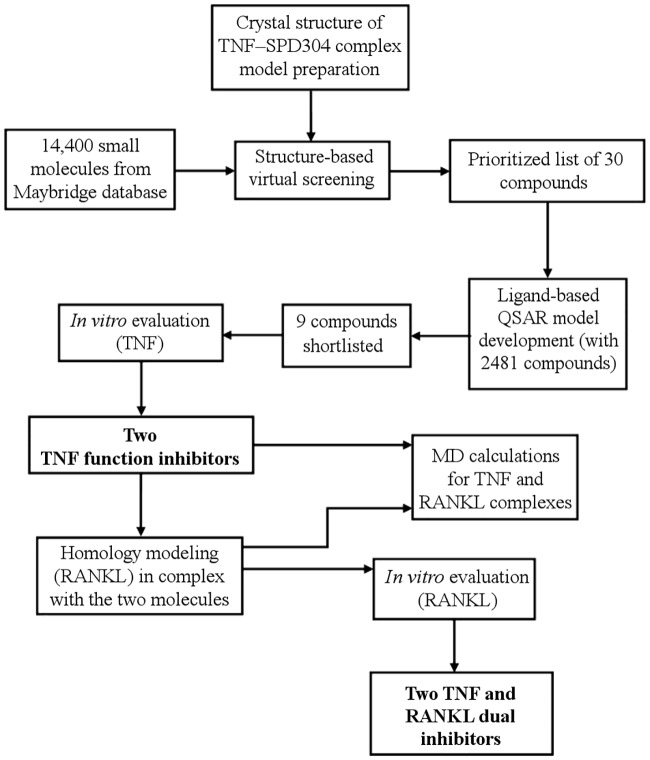
Strategy for the identification of new small-molecule PPI inhibitors for TNF and RANKL.

In summary, our strategy for identifying the new dual TNF and RANKL inhibitors, T8 and T23, is shown below (Fig 2):

All steps, including structure–and ligand–based modeling, *in vitro* evaluation and molecular dynamics calculations, are discussed below.

### Virtual screening and molecular docking calculations

As a first step in our pipeline, all 14,400 compounds included in the Maybridge database were *in silico* investigated in a structure-based approach using docking and molecular modeling approaches. All the compounds of the abovementioned database passed the Lipinski “rule of five” for drug likeness and are non-reactive, ensuring fewer false positives and higher quality results. The crystal structure of TNF dimer in complex with inhibitor SPD304 (PDB code: 2AZ5)[27] was used as the molecular template for our investigation. Each compound was docked into the active site of the protein using the Surflex-Dock module of SYBYL 8.0 suite.[44] Based on the docking scores of the ligand poses, we generated a prioritized list of 30 compounds for further consideration. Detailed information on the molecular modeling methodology is presented in the Supporting Information (S1 Text).

### Ligand–based predictive model development

A ligand–based model was developed to complement our methods in an effort to identify the most promising compounds among those proposed in the previous structure–based approach. Our model was built based on the KNIME platform with the help of our in-house developed Enalos KNIME nodes.[45]

#### Data set

The model was based on the largest available dataset of TNF inhibitors, including 2481 compounds that were retrieved from the PubChem database and had been tested in an HTS assay for NF-κB induction by TNF. TNF is one of the key cytokines that initiate the signaling cascade that leads to NF-κB induction[40]. Among the available compounds, the 1149 that exhibited inhibition more than 50% were characterized as active and the remaining 1332 were characterized as inactive.[40]

#### Descriptor calculation–training and test set separation

For each compound included in the dataset, 777 descriptors were calculated using the Mold2 software[46] through the Enalos Mold2 KNIME node.[45] After normalizing the data and applying the low variance filter, 616 descriptors remained. For validation purposes, our original dataset was also divided into training and test sets using the Fuzzy c-Means clustering approach included in KNIME [47]. First, the dataset was divided into 5 clusters and within each cluster, compounds were divided into training and test set in a ratio of 80:20. Training and test compounds from all clusters were then compiled and in total 1985 compounds constituted the training set and 495 the test set.

#### Modeling and validation

KNIME analytics platform[48] offers the opportunity to experiment with a large number of methodologies simultaneously and to explore which best fits our data. After doing so, we concluded in a consensus approach that outperformed all other methodologies tested. We built three different models based on the k-Nearest Neighbor, the Nearest Neighbor and Random Forest. For each methodology used, we selected different variable selection methods, including GainRatioAttribute Evaluator combined with Ranker search method, CfsSubset Evaluator combined with Best First search method and InfoGainAttribute Evaluator combined with Best First search method, respectively, for each of the aforementioned models. Details on these methodologies can be found in the literature.[49,50] On top of these models, a consensus model was also built based on majority vote from each of the individual models. This model outperformed all others based on validation metrics that were used to assess the predictive power of each model, namely specificity, sensitivity, accuracy and precision. Confusion matrix is also provided for each methodology tested. Details on these metrics are given in the Supporting Information (S1 Text).

#### Domain of applicability

Our developed model aspires to emerge as a useful tool for the virtual screening of newly introduced small molecules not included in our original dataset. In this virtual screening framework,[51] the reliability of provided predictions needs to be assessed and thus it is important that the domain of applicability of the model is well defined. When the model’s applicability limits are known, predictions for new molecular entries can be highlighted as reliable or not.

For the developed model, in order to define the domain of applicability, Euclidean distances between each compound in the test set and its nearest neighbor in the training set are calculated and then compared to a calculated threshold. For a distance greater than the threshold the prediction is considered unreliable. Details on the domain of applicability calculation are given in the literature.[52–57] To implement the domain of applicability calculation, we used the Enalos Domain–Similarity node.

### Enalos cloud platform

The Enalos Cloud Platform[58,59] is a platform that hosts several predictive models for drug discovery and risk assessment of small molecules and nanoparticles.[60] In the previous steps, we succeeded to develop a predictive consensus model for TNF inhibition. Since, to the best of our knowledge, this is the only ligand–based model developed from an extensive dataset of TNF inhibitors, we decided to release it as a web service to facilitate the need for the design and virtual screening of novel potent small–molecule inhibitors of TNF, by providing immediate access to the model’s results. The model can be easily accessed through http://enalos.insilicotox.com/TNFPubChem. The interested user can initiate a prediction through a user friendly graphical interface following a minimum-step procedure. The user can submit a structure by using one of the following ways: (i) draw a structure using the sketcher provided in the first page,[61] (ii) submit a SMILES notation of a molecule, or (iii) upload an sdf file. In any case, more than one compound can also be submitted. When the “TNFPubChem” workflow is selected, the compounds are submitted and the prediction is generated. The results page includes the predicted classification and an indication based on the domain of applicability on whether the provided prediction can be considered reliable or not. Screenshots of this online tool are shown and discussed in the Results and Discussion Section.

### TNF–induced death assay in L929 cells[62]

L929 cells were cultured on 96-well plates (3×10^4^ cells/well) overnight. Then, 0.25 ng/mL human TNF (PeproTech) and 2 μg/mL actinomycin D (Sigma–Aldrich) were added. TNF with the compounds were pre-incubated for 30 min at room temperature before adding them to cells to assess inhibition. Background death was estimated using an actinomycin D treated-only control. After approximately 24 h, dead cells were cleared away by washing with PBS. The remaining live cells were then fixed with methanol and stained with crystal violet. After solubilization with acetic acid, staining was quantified spectrophotometrically at 570 nm. Cytotoxicity is expressed with respect to the background death control and also relative to the toxicity of compounds. Experiments were repeated three times.

### Measurement of cytotoxicity in L929 cells[62]

L929 cells were seeded onto a 96-well plate (3x10^4^ cells/well). After 24 h, cells were treated with the compounds at increasing concentrations. DMSO was used in the untreated control. The next day, a PBS-washing step was performed to remove dead cells. The remaining live cells were next fixed with methanol and stained with crystal violet. After solubilization with acetic acid, staining was quantified spectrophotometrically at 570 nm. Survival is expressed with respect to the untreated control. Experiments were performed in triplicate.

### TNF/TNFR1 ELISA assay[62]

96-well plates were coated with 0.1 mg/mL recombinant soluble human TNFR1 (PeproTech) in PBS overnight at 4°C. Next, blocking was performed with 1% BSA in PBS after carrying out four washes with PBS containing 0.05% Tween-20. 0.025 mg/mL recombinant human TNF (PeproTech) in PBS was added and the plates were incubated for 1 h at room temperature. After two consecutive rounds of washes, plates were incubated with a rabbit anti-human-TNF antibody (provided by Prof. W. A. Buurman, University of Maastricht) and an anti-rabbit secondary antibody conjugated with HRP (Vector Laboratories). Both incubations were for 1 h and were performed at room temperature. Following a last round of washes, the signal was finally developed using the TMB Substrate Kit (ThermoFisher Scientific) and was measured spectrophotometrically at 450 nm. Experiments were performed in triplicate.

### RANKL-induced osteoclastogenesis assay

Bone marrow cells (BMs) were collected after flushing out of femurs and tibiae, subjected to gradient purification using ficoll-paque (GE Healthcare), plated on 96-well plates at a density of 6 × 10^4^ cells per well in αMEM (GIBCO) containing 10% fetal bovine serum supplemented with 50 ng/ml human RANKL, 25 ng/ml M-CSF[42] in the presence or absence of T8 and T23 compounds in the indicated concentrations (1, 2, 5, 10 and 20 μM) for 5 days. To visualize osteoclasts, cell cultures were stained with TRAP (tartrate-resistant acid phosphatase), using an acid phosphatase leukocyte (TRAP) kit (Sigma-Aldrich). Osteoclasts were identified as TRAP-positive multinucleated cells containing more than three nuclei.

### Osteoclastic (TRAP) activity assay

To measure osteoclast activity, bone marrow cells were cultured for four days as described above and lysed with 0.2% Triton X-100. TRAP activity was measured by the conversion of p-nitrophenyl phosphate to p-nitrophenol in the presence of sodium tartrate. Thus, cell lysates were incubated with 5.5 mM phosphatase substrate diluted in 100 mM citrate buffer and 10 mM sodium tartrate, for 40 minutes at 37°C. The reaction was stopped by addition of 0.4 N sodium hydroxide and absorbance was measured at 405 nm using a microplate reader (Molecular Devices). TRAP activity was normalized to total protein content as determined by the Bradford assay (Bio-Rad protein assay). TRAP activity per μg of total protein was expressed as a percentage of the positive control treated without compounds. IC_50_ values were calculated as mean ± SEM from three independent experiments performed in duplicate.

### BM viability

BMs were plated on 96-well plates at a density of 10^5^ cells/well and treated with T8 and T23 at various concentrations, in the presence of M-CSF (25 ng/mL) for 48 hours. Cell viability was determined by the 3-(4,5-dimethylthiazol-2-yl)-2,5-diphenyltetrazolium bromide (MTT) colorimetric assay, which measures the ability of viable cells to reduce a soluble tetrazolium salt to an insoluble purple formazan precipitate.[63] After removal of the medium, each well was incubated with 0.5 mg/mL MTT (Sigma-Aldrich, St. Louis, MO) in serum-free α-MEM at 37°C for 3 h. At the end of the incubation period, the medium was removed and the intracellular formazan was solubilized with 200 μL DMSO and quantified by reading the absorbance at 550 nm on a microplate reader (Optimax; Molecular Devices, Sunnyvale, CA). Cell viability (%) was expressed as a percentage of the negative control treated without compounds. LC_50_ values were calculated as mean ± SEM from three independent experiments.

### RANKL homology modeling

The homology structure of RANKL in complex with SPD304 was constructed based on the mouse RANKL crystal structure (PDB code: 1S55) and the TNF dimer with SPD304 (PDB code: 2AZ5) using the jFATCAT pairwise structure alignment algorithm.[64] The derived structure was used for the subsequent MD calculations.

### Molecular dynamics calculations

Unrestrained MD simulations in water were performed for TNF and RANKL complexes with compounds T8 and T23 using AMBER 12.[65,66] Water molecules were discarded from the crystal structures and hydrogen atoms were added to the proteins with AMBER. TNF and RANKL missing residues were included in the crystal structures with Modeller 9.10.[67,68] Atomic partial charges, bonded and non-bonded parameters for the proteins were represented by the modified ff99SB force field.[69] Geometry optimization of the compounds was obtained with Gaussian 09[70] at the HF/6-31G* level and the ANTECHAMBER program was employed to derive the RESP atomic partial charges for T8 and T23. Force field parameters for T8 and T23 were represented by the general AMBER force field (GAFF).[71] The complexes were neutralized by adding 4 Na^+^ (TNF complexes) and 7 Cl^−^ (RANKL complexes) counterions. TIP3P water molecules[72] were used to model explicit solvation in a truncated octahedron, with a cutoff 10 Å. Long-range electrostatics were considered by the particle mesh Ewald (PME) approach.[73] A combination of lengthy, multi-step steepest descent and conjugate gradient iterations were performed to achieve thorough energy minimization of the complexes: initially, a 500 kcal mol^−1^ Å^−2^ restraint was applied to the solute (ligand−protein) to allow minimization of the positions of water molecules only. Then, minimization continued for two more steps with diminished restraints on the solute (10 and 2 kcal mol^−1^ Å^−2^), before totally removing the restraint for the last step of the minimization. Each minimization stage was carried out in 5000 cycles with a 20 Å cutoff. Subsequently, the systems were gently heated in the NVT ensemble for 100 ps, from 0 K until the target temperature of 310 K was reached. Hydrogen motion was not included in the calculations by applying the SHAKE algorithm[74], thus allowing a 2 fs time step to be used. The Langevin thermostat[75] (collision frequency = 2.0 ps^−1^) was employed to control the temperature. During heating, the solute was also moderately restrained (force constant 10 kcal mol^−1^ Å^−2^). This restraint was kept for the subsequent 100 ps of equilibration in constant pressure. A final unrestrained MD equilibration step of 100 ps was carried out for each complex. Constant-pressure MD simulations for 50 ns were produced with the GPU (CUDA) version of PMEMD in AMBER 12.[76] The same procedure was applied for two additional simulations, namely for complexes SPD304–TNF and SPD304–RANKL to compare our results with those of the known TNF inhibitor. Trajectory analysis (RMSD, atomic fluctuations, and hydrogen bond calculations) was performed with the ptraj module of AMBER. Donor−acceptor distance and donor−hydrogen−acceptor angle cutoffs of 3.5 Å and 120°, respectively, were used to calculate hydrogen bond (HB) interactions.

### MM–PBSA free energy calculations

The Molecular Mechanics Poisson-Boltzmann Surface Area (MM−PBSA) method estimates free energies of (bio)molecular systems in solution by performing end-state calculations.[77–80] The division of the binding free energy into individual components offers additional information regarding complex formation. For an inhibitor–protein complex, the following process directs the binding free energy change (Δ*G*_bind_):

Protein+Inhibitor→Complex

Calculations were performed on 5000 frames from each trajectory after all water molecules and counterions were removed. For every snapshot, the binding energy is calculated with the following equation:

$$\Delta G_{\text{bind}}=G_{\text{complex}}\;-\;(G_{\text{protein}}+\;G_{\text{inhibitor}}),$$ (1)

where Δ*G*_bind_ is the total binding free energy, *G*_complex_, *G*_protein_, and *G*_inhibitor_ are the energies for the complex, the protein (TNF or RANKL), and the inhibitor (T8, T23, SPD304), respectively. The binding free energy can be divided into enthalpy and entropy contributions:

$$\Delta G_{\text{bind}}=\Delta H\;-\;T\Delta S$$ (2)

The enthalpy is given by

$$\Delta H=\Delta E_{\text{MM}}\;+\;\Delta G_{\text{sol}},$$ (3)

where Δ*E*_MM_ is the interaction energy between the protein and the inhibitor and is approximated with the molecular mechanics (MM) method, while Δ*G*_sol_ defines the change in the free energy of solvation upon ligand binding. Δ*E*_MM_ is further separated into:

$$\Delta E_{\text{MM}}=\Delta E_{\text{elec }}+\;\Delta E_{\text{vdW}}$$ (4)

Δ*E*_elec_ is the electrostatic interaction energy and Δ*E*_vdW_ is the van der Waals interaction energy; no cutoff was applied for the calculation of these two terms. Moreover, the solvation energy (Eq 3) may be expressed in electrostatic (Δ*G*_PB_) and nonpolar (Δ*G*_NP_) terms:

$$\Delta G_{\text{sol}}=\Delta G_{\text{PB}}\;+\;\Delta G_{\text{NP}}$$ (5)

The electrostatic (Δ*G*_PB_) energy is estimated by the Poisson−Boltzmann (PB) approach[81] through the PBSA module of AMBER, and a solvent-accessible surface area (SASA) term is used to express the hydrophobic contribution to solvation (Δ*G*_NP_):

$$\Delta G_{\text{NP}}=\gamma *\text{SASA }+\;\beta$$ (6)

For the surface tension (γ) and the offset (β), the default values of 0.005420 kcal mol^−1^ Å^−2^ and −1.008000 kcal mol^−1^, respectively, were considered. A probe radius of 1.4 Å was applied for the solvent (water) in the SASA calculation. Δ*G*_NP_ (Eq 6) was computed with the linear combinations of pairwise overlaps (LCPO) method.[82] The values of solvent and solute dielectric constants were fixed at 80.0 and 1.0, respectively.[83] The finite-difference grid spacing was 0.5 Å and the ratio between the longest dimension of the grid and that of the solute was set to 4.0. The ionic strength was considered to be 0.1 M. The contribution of entropy (Eq 2) was calculated with the nmode routine of AMBER 12 over the last 100 snapshots of the trajectories, for computational efficiency.

### Cross-linking experiments

100 ng of recombinant human TNF or RANKL (Peprotech) were incubated with the inhibitors for 30 min at room temperature and then subjected to cross-linking, using 4.8 mM BS3 (ThermoFisher Scientific) for 30 min at room temperature. The reaction was stopped by adding 1/10th volume of 1 M Tris-HCl, pH 7.5. Samples were then subjected to SDS-PAGE and western blotting using an anti human-TNF or RANKL antibody (both R&D Systems).

### Binding affinity assay

For use in the binding assays, recombinant TNF and RANKL were expressed and purified as previously described.[84–86] Fluorescence intensity was measure with a Synergy H1 Multi-Mode Reader (BioTek) in 96-well microplates (black) at 25°C. Briefly, a series of solutions (0.1 mL) containing either TNF (0.75 μM) or RANKL (0.5 μM) and increasing amounts of the ligands were prepared. During preparation of the solutions the buffer was firstly added to all wells followed by the ligand solution and finally the protein was added. All solutions were mixed by pipetting and equilibrated for 1h at 25°C before measurement. Differences in fluorescence intensity values (λex = 274 nm; λem = 302 nm) between the various protein/ligand complexes and free protein were fitted on a second order equation as described in the Supporting Information (S2 Text) in order to determine the dissociation constant (K_d_) of TNF and RANKL with SPD304, T23 and T8. Experiments were performed for TNF in 10 mM citrate-phosphate (pH 6.5) and for RANKL in 25 mM Tris–HCl buffer, 100 mM NaCl (pH 7.5); both buffers were supplemented with PEG3350 at the concentrations indicated in the respective figures. K_d_ values were calculated as mean ± SEM from three independent experiments.

## Results and discussion

We initiated our cheminformatics-aided workflow for the identification of novel TNF inhibitors with an *in silico* approach based on the TNF crystal structure in complex with SPD304 and a pool of small molecules included in the Maybridge Hitfinder database. The biologically-active TNF is a trimer of identical subunits and SPD304 has been reported to displace one of the subunits, thus resulting in inactive species; that means that functional inhibition is affected by obstructing trimerization. [22,27] The binding site of TNF dimer in this protein-protein interaction is characterized as predominantly hydrophobic, consisting of glycine, leucine and tyrosine residues. Reported interactions with small molecules are described as hydrophobic and shape-driven as the molecular structures need to be large enough to interact with both subunits to prevent the binding of the third subunit to the TNF dimer. All the 14400 small molecules included in the Hitfinder database were *in silico* explored in a structure-based framework using molecular modeling and docking scoring. The crystal structure of TNF dimer with SPD304 (PDB code: 2AZ5) was used as the molecular model for our investigation and the compounds were docked into the enzyme’s active site. The molecular docking studies were performed using the Surflex-Dock algorithm of SYBYL.[44] Based on the structure-based results, a prioritized list of 30 compounds was constructed by a combination of high docking scores with optimal placements into the binding cavity that resemble the SPD304 binding arrangement for further *in silico* screening with a ligand-based developed model. The ranking of these prioritized 30 compounds is given in Table 1 and in Supporting Information (S1 Table).

**Table 1:** Ligand–based predictions through the Enalos cloud platform (http://enalos.insilicotox.com/TNFPubChem/), Tanimoto similarity to SPD304, PAINS check.

ID	InChI	Consensus Prediction	Domain of Applicability	Tanimoto Similarity to SPD304	PAINS[87]
SPD304	InChI = 1S/C32H32F3N3O2/c1-21-14-28-30(15-22(21)2)40-20-24(31(28)39)18-37(4)13-12-36(3)17-23-19-38(29-11-6-5-10-27(23)29)26-9-7-8-25(16–26)32(33,34)35/h5-11,14–16,19-20H,12–13,17-18H2,1-4H3	Active	Reliable	-	No
1	InChI = 1S/C28H33N3O4S/c32-26-21-28(18-17-22-9-3-1-4-10-22,27(33)31(26)24-11-5-2-6-12-24)29-23-13-15-25(16-14-23)36(34,35)30-19-7-8-20-30/h1,3–4,9–10,13–18,24,29H,2,5–8,11–12,19-21H2/b18-17+/t28-/m0/s1	Active	Reliable	0.29	No
2	InChI = 1S/C27H29NO4/c1-20(28-26(29)18-21-9-5-3-6-10-21)13-14-23-15-16-24(25(17–23)31-2)32-27(30)19-22-11-7-4-8-12-22/h3-12,15–17,20H,13–14,18-19H2,1-2H3,(H,28,29)/t20-/m1/s1	Active	Reliable	0.34	No
3	InChI = 1S/C25H15F3N2O6/c26-25(27,28)16-12-13-20(19(15–16)30(32)33)35-21-10-4-5-11-22(21)36-24(31)18-9-6-14-29-23(18)34-17-7-2-1-3-8-17/h1-15H	Active	Reliable	0.42	No
4	InChI = 1S/C26H28FN3O4/c1-31-23-9-8-17(13-24(23)32-2)12-22-21-16-26(34–4)25(33–3)14-18(21)10-11-30(22)29-28-20-7-5-6-19(27)15-20/h5-9,13–16,22H,10-12H2,1-4H3/b29-28+/t22-/m0/s1	Inactive	Reliable	0.31	Yes
5	InChI = 1S/C21H19Cl3N4O3/c1-12-20(30-18-5-3-15(22)4-6-18)13(2)28(26–12)8-7-19(25)27-31-21(29)14-9-16(23)11-17(24)10-14/h3-6,9-11H,7-8H2,1-2H3,(H2,25,27)/p+1	Inactive	Reliable	0.28	No
6	InChI = 1S/C28H35NO8S2/c1-6-35-28(36-7-2)20-29(38(30,31)24-13-8-21(3)9-14-24)19-23-12-17-26(34–5)27(18–23)37-39(32,33)25-15-10-22(4)11-16-25/h8-18,28H,6–7,19-20H2,1-5H3	Inactive	Reliable	0.26	No
7	InChI = 1S/C19H25N5O4/c1-13(25)21-14-6-4-7-15(10–14)28-9-5-8-24-11-16-17(20-12-24)22(2)19(27)23(3)18(16)26/h4,6–7,10,20H,5,8–9,11-12H2,1-3H3,(H,21,25)/p+1	Inactive	Reliable	0.32	No
8	InChI = 1S/C22H17BrO4/c1-25-18-11-10-15(23)12-14(18)13-26-22(24)21-16-6-2-4-8-19(16)27-20-9-5-3-7-17(20)21/h2-12,21H,13H2,1H3	Inactive	Reliable	0.3	No
9	InChI = 1S/C25H27NO2/c1-19-10-13-24-21(15–19)9-6-14-26(24)17-22-11-12-23(16-25(22)27-2)28-18-20-7-4-3-5-8-20/h3-5,7–8,10–13,15-16H,6,9,14,17-18H2,1-2H3	Inactive	Reliable	0.37	No
10	InChI = 1S/C20H29NO12/c1-11(22)29-10-16(30-12(2)23)17(31-13(3)24)18(32-14(4)25)19(33-15(5)26)20(27)21-6-8-28-9-7-21/h16-19H,6-10H2,1-5H3/t16-,17-,18+,19-/m1/s1	Active	Unreliable	0.22	No
11	InChI = 1S/C21H25N3O9S/c1-4-30-21(25)14-12-17(31-10-8-28-2)18(32-11-9-29-3)13-16(14)24-34(26,27)19-7-5-6-15-20(19)23-33-22-15/h5-7,12–13,24H,4,8-11H2,1-3H3	Inactive	Reliable	0.29	Yes
12	InChI = 1S/C28H26ClN3O2/c1-18-26(27(31-34-18)22-12-6-7-13-23(22)29)28(33)30-24-14-8-4-10-20(24)17-25-21-11-5-3-9-19(21)15-16-32(25)2/h3-14,25H,15-17H2,1-2H3,(H,30,33)/t25-/m0/s1	Active	Reliable	0.29	No
13 (T8)	InChI = 1S/C26H22ClF3N6O3S2/c27-18-7-5-17(6-8-18)24(37)31-32-25(35-13-1-2-14-35)34-41(38,39)20-11-9-19(10-12-20)36-21(22-4-3-15-40-22)16-23(33–36)26(28,29)30/h3-12,15–16,25,34H,1–2,13-14H2/b32-31+/t25-/m0/s1	Active	Reliable	0.3	No
14	InChI = 1S/C20H20N2O5S3/c23-29(24,16-6-2-1-3-7-16)19-10-11-20(28–19)30(25,26)21-17-8-4-5-9-18(17)22-12-14-27-15-13-22/h1-11,21H,12-15H2	Inactive	Reliable	0.2	No
15(T23)	InChI = 1S/C22H22O10/c1-11(23)29-17-9-19(27–5)21(31-13(3)25)7-15(17)16-8-22(32-14(4)26)20(28–6)10-18(16)30-12(2)24/h7-10H,1-6H3	Active	Reliable	0.25	No
16	InChI = 1S/C27H19ClN8/c28-21-13-11-19(12-14-21)25-20(17-35(34–25)22-7-3-1-4-8-22)15-31-33-26-24-16-32-36(27(24)30-18-29-26)23-9-5-2-6-10-23/h1-14,16-18H,15H2/b33-31+	Active	Reliable	0.24	No
17	InChI = 1S/C27H26N2O5/c1-18(30)29-26(21-13-15-22(32–3)16-14-21)27(34-19(2)31)25(28–29)23-11-7-8-12-24(23)33-17-20-9-5-4-6-10-20/h4-16,26-27H,17H2,1-3H3/t26-,27-/m0/s1	Inactive	Reliable	0.33	No
18	InChI = 1S/C19H36N4OS/c1-3-4-5-6-7-8-9-10-11-12-13-14-15-20-18(24)16-25-19-22-21-17-23(19)2/h17H,3-16H2,1-2H3,(H,20,24)	Active	Reliable	0.15	No
19	InChI = 1S/C22H23NO11/c1-10(24)30-9-16-18(31-11(2)25)19(32-12(3)26)17(22(34–16)33-13(4)27)23-20(28)14-7-5-6-8-15(14)21(23)29/h5-8,16–19,22H,9H2,1-4H3/t16-,17-,18-,19-,22-/m1/s1	Active	Unreliable	0.29	No
20	InChI = 1S/C25H26F3N3O2/c1-32-23-15-22(33-18-19-5-3-2-4-6-19)9-7-20(23)17-30-11-13-31(14-12-30)24-10-8-21(16-29-24)25(26,27)28/h2-10,15-16H,11–14,17-18H2,1H3	Inactive	Reliable	0.45	No
21	InChI = 1S/C20H21N3O4S/c1-14-17(20(22-27-14)15-6-4-3-5-7-15)13-21-28(24,25)16-8-9-19-18(12–16)23(2)10-11-26-19/h3-9,12,21H,10–11,13H2,1-2H3	Inactive	Reliable	0.3	No
22	InChI = 1S/C26H30N4O2S2/c1(3-11-17-23-27-29-25(31–23)33-19-21-13-7-5-8-14-21)2-4-12-18-24-28-30-26(32–24)34-20-22-15-9-6-10-16-22/h5-10,13-16H,1–4,11–12,17-20H2	Inactive	Reliable	0.15	No
23	InChI = 1S/C24H26N4O4S2/c1-17-7-9-19(10-8-17)34(31,32)28-13-11-27(12-14-28)16-22(29)26-20-15-21(33-23(20)24(25)30)18-5-3-2-4-6-18/h2-10,15H,11–14,16H2,1H3,(H2,25,30)(H,26,29)	Inactive	Reliable	0.24	No
24	InChI = 1S/C23H18N6OS/c1-16-22(28-12-6-5-11-20(28)25-16)23(30)26-24-14-17-15-29(18-8-3-2-4-9-18)27-21(17)19-10-7-13-31-19/h2-15H,1H3,(H,26,30)/b24-14-	Inactive	Reliable	0.26	No
25	InChI = 1S/C22H27NO11/c1-12(24)30-11-18(31-13(2)25)19(32-14(3)26)20(33-15(4)27)21(34-16(5)28)22(29)23-17-9-7-6-8-10-17/h6-10,18-21H,11H2,1-5H3,(H,23,29)/t18-,19-,20+,21-/m0/s1	Active	Unreliable	0.25	No
26	InChI = 1S/C18H20FNO4S/c1-23-17-11-8-14(12-18(17)24-15-4-2-3-5-15)20-25(21,22)16-9-6-13(19)7-10-16/h6-12,15,20H,2-5H2,1H3	Inactive	Reliable	0.24	No
27	InChI = 1S/C25H27NO2S/c1-28-24-12-5-4-11-23(24)20-13-15-26(16-14-20)25(27)18-29-17-21-9-6-8-19-7-2-3-10-22(19)21/h2-12,20H,13-18H2,1H3	Inactive	Reliable	0.36	No
28	InChI = 1S/C17H16N4O/c22-17(11-21-16-8-4-3-7-15(16)19-20-21)18-14-9-12-5-1-2-6-13(12)10-14/h1-8,14H,9-11H2,(H,18,22)	Inactive	Reliable	0.28	No
29	InChI = 1S/C25H32N2O2/c1-3-29-25(28)20-12-15-26(16-13-20)23-9-5-4-7-22(23)18-27-14-6-8-21-17-19(2)10-11-24(21)27/h4-5,7,9–11,17,20H,3,6,8,12–16,18H2,1-2H3	Inactive	Reliable	0.32	No
30	InChI = 1S/C17H21N5O3S2/c23-27(24,15-8-2-1-3-9-15)21-16(22-10-4-5-11-22)19-20-17(26)18-13-14-7-6-12-25-14/h1-3,6–9,12,16,21H,4–5,10–11,13H2,(H,18,26)/b20-19+/t16-/m0/s1	Active	Reliable	0.22	No

After the initial structure-based screening of our original datasets, we filtered out less promising compounds by developing and applying a ligand-based predictive model. Our model was built on a set of almost 2500 compounds that are included in the PubChem database and have been identified as TNF inhibitors based on an *in vitro* HTS assay.[40] The ligand-based model was implemented through the KNIME platform[48] with the aid of our in-house developed Enalos KNIME nodes[45] that execute several tasks that are crucial for model development. In particular, we used the Enalos Mold2 node that calculates hundreds of molecular descriptors to encode structural information of compounds, and the Enalos Domain of applicability that is used to define the area of reliable predictions. Details on the Mold2 descriptors are provided in the Supporting Information (S1 Text and S2 Table).

Since the developed KNIME workflow allows this flexibility, we experimented with a great variety of variable selection and model development methods. Moreover, we experimented with consensus approaches and concluded in the most accurate and predictive model, which is a consensus model based on the majority vote of the results of three different modeling methodologies, namely k-Nearest Neighbor, Nearest Neighbor and Random Forest.[49]

The proposed models were validated both internally and externally in terms of goodness-of-fit, robustness and predictivity and were proven to successfully fulfill the criteria recommended by the Organization for Economic Cooperation and Development (OECD) for model validation.[88] For validation purposes, the dataset was separated into training and test sets. The small molecules included in the latter were kept as a blind set and were not used during the development of the model.

The validation results for each methodology and the consensus model are shown in Tables 2 and 3. Based on these metrics, the consensus model outperformed the individual models that were built and thus was considered as the most accurate and reliable to be used in our virtual screening process.

**Table 2:** Validation results for the test set.

	Precision	Sensitivity	Specificity	Accuracy
**kNN**	0.686	0.665	0.736	0.703
**Nearest Neighbor**	0.704	0.704	0.743	0.725
**Random Forest**	0.653	0.630	0.709	0.673
**Consensus**	**0.747**	**0.717**	**0.789**	**0.756**

**Table 3:** Confusion matrix.

**kNN**	Positive Predicted (Active)	Negative Predicted (Inactive)
Positive Observed (Active)	153	77
Negative Observed (Inactive)	70	195
**Nearest Neighbor**	Positive Predicted (Active)	Negative Predicted (Inactive)
Positive Observed (Active)	162	68
Negative Observed (Inactive)	68	197
**Random Forest**	Positive Predicted (Active)	Negative Predicted (Inactive)
Positive Observed (Active)	145	85
Negative Observed (Inactive)	77	188
**Consensus**	Positive Predicted (Active)	Negative Predicted (Inactive)
Positive Observed (Active)	165	65
Negative Observed (Inactive)	56	209

A crucial aspect, which is often neglected by similar studies presented in the literature, is the dissemination of the results to the wider community.[89] It is of major importance that the developed model is not retained only for developers’ use, but is broadly distributed to the scientific community so that it could serve as a direct source of information. Furthermore, as recently highlighted by several initiatives, this is most effectively achieved by providing open source tools that could be easily implemented and adjusted to the distinctive requirements of each project.

**Fig 3:**
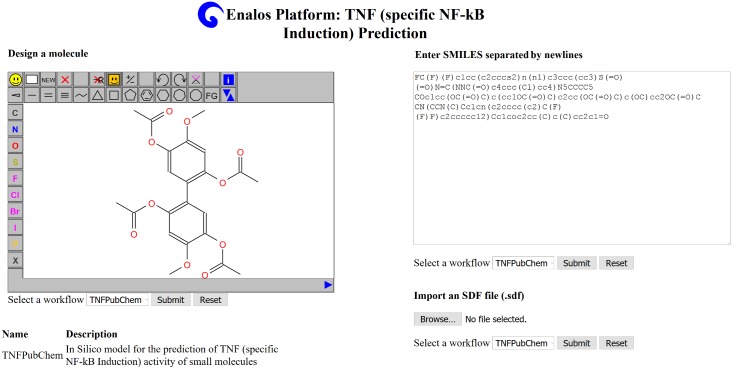
The sketching and structural modification facility for the prediction of new TNF inhibitors as provided by the Enalos cloud platform (screen shot).

To encourage and facilitate the reuse of our predictive model, the consensus model has been made publicly available online via the Enalos Cloud Platform. Our model is hosted under the following url: http://enalos.insilicotox.com/TNFPubChem/ and is easily accessible online from any browser, also supporting mobile devices. The interested user can make their own predictions using the user friendly graphical interface that allows multiple options for submitting a new structure. First, as seen in the screen shot below (Fig 3), a sketcher is available where a new molecule can be drawn and structurally modified. The structure can be either directly submitted to generate a prediction or can be copied as SMILES. The second option includes the SMILES notation submission in the upper right of the web page, where the user can paste one or more SMILES notations for one or a batch of molecules and then submit the whole list for prediction. Finally, with the third option the user can upload an SDF file with multiple entries and submit the file for prediction.

**Fig 4:**
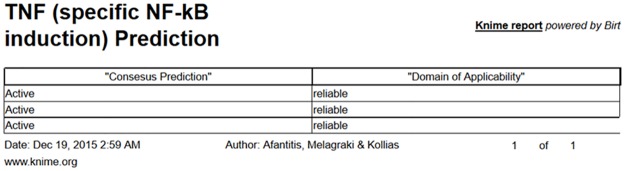
Prediction and reliability results regarding TNF inhibition upon structure submission of T23, T8 and SPD304 (see SMILES Fig 3) to the Enalos cloud platform.

When the model “TNFPubChem” is selected and the structures are submitted in either way, the prediction is generated as an html page or a CSV file. The prediction outcome includes a classification for each of the given structures and an indication of whether the prediction can be considered reliable or not, based on the domain of applicability of the model (Fig 4). The web service does not require special computational skills and can be easily used by scientists of different disciplines, including chemists, biologists, physicists, and engineers, involved or interested in the biological evaluation of TNF inhibitors.

The consensus model was finally used to predict TNF inhibition by the 30 compounds proposed in the previous step. Among those, the top 9 commercially-available small molecules being predicted as active by the ligand-based model within its domain of applicability (Table 1) were selected for experimental validation to quantify their inhibitory potency against TNF. These computationally-identified hits were also filtered for Pan Assay Interference Compounds (PAINS).[87,90] As shown in Table 1, none of the predicted active compounds was identified as PAINS and thus none was excluded from the subsequent experimental validation. Moreover, we tested whether a simpler approach using 2D similarity search based on Tanimoto metric between the known active compound (SPD304) and our identified hits would have yielded comparable results with our proposed methodology. Low similarity between SPD304 and identified hits in the range of 0.15–0.45 (Table 1) confirmed that a simpler approach could not have proposed the structures identified based on our methodology.

Biological screening of the compounds examined their capacity to block TNF function in a modified TNF bioactivity assay.[91] The basis of this test is the death-inducing function of TNF in the murine fibrosarcoma cell line L929 following sensitization by the transcription inhibitor actinomycin D. Functional potency of the compounds would translate in a significant reduction of TNF-induced death. Using this approach, out of the nine shortlisted small molecules, two compounds (T8 and T23) were selected as follows. After an initial screening at a concentration of 20 μM, compounds displaying ≥ 25% inhibition of TNF-induced cell death were further examined in dose-response tests in order to estimate their IC50 values. In these dose-response experiments, T8 and T23 inhibited TNF-driven toxicity in L929 cells with IC_50_ values of 40±2.3 and 17±1.2 μM, respectively (S1 Fig). Given that screening has been based on protection from TNF-induced death, compounds that were highly toxic themselves would not have passed this first level of testing. Indeed, both compounds were found to have low toxicity against L929 cells (S2 Fig). Importantly, NMR and MS data for T8 and T23, as shown in S3–S6 Figs and S2 Text in Supporting Information, confirmed the purity for both compounds to be above 95%.

Having established that the selected compounds obstruct TNF function, and since TNF exerts its functions mainly through interacting with the TNFR1 receptor, a further test was performed to evaluate the effect of inhibition on this protein-receptor interaction. The ELISA-based test revealed that both compounds significantly block this interaction, with measured IC_50_ values of 30±2.3 (T8) and 3±0.1 (T23) μM (S7 Fig).

Finally, direct binding of the compounds to TNF was demonstrated using a fluorescence-based binding assay (S8 Fig). T23 was found to have the lowest K_d_ value (2.8±0.2 μM), while T8 had a K_d_ of 8.8±0.8 μM, and SPD304 of 5.8±0.5 μM.

In previous work, we had shown that SPD304 is also a potent inhibitor of RANKL[42], another member of the TNF superfamily, mainly involved in the regulation of osteoclast formation and bone resorption.[92] We were hence prompted to explore the two compounds with respect to RANKL also, firstly by *in vitro* testing. We evaluated the effect of T8 and T23 on RANKL-dependent osteoclast differentiation in a culture system of bone marrow cells stimulated with RANKL and M-CSF for 5 days through evaluation of the tartrate-resistant acid phosphatase (TRAP) activity, an osteoclast-specific enzyme. Using a quantitative assay that measures TRAP activity, both compounds were found to inhibit significantly the formation of osteoclasts in a dose-dependent manner, with an IC_50_ of 9.0±0.7 μM (T8) and 2.6±0.2 μM (T23) (S9 Fig). In order to exclude the possibility that the inhibitory effect of the compounds is correlated with high cell toxicity, a viability assay was employed in primary bone marrow cells. Of the two compounds, T8 displayed a low toxicity with an LC_50_ of 42.6±4.3 μM, while T23 had a higher toxicity (LC_50_ = 5.9±0.3 μM, S10 Fig). It should be noted that the increased toxicity of the two compounds in these cells in comparison to the toxicity in L929 cells could be due to the increased sensitivity of primary cells and that in both cases, the toxicity was lower as compared to SPD304. Direct binding of the compounds to RANKL was validated with K_d_ values of 6.3±0.6 μM (T8) and 7.3±0.4 μM (T23) (S11 Fig). Interestingly, these values were lower than that of SPD304, which was measured to be 13.8±0.7 μM. All *in vitro* results for both TNF and RANKL are shown in Table 4.

**Table 4:** Inhibition, toxicity and binding evaluation of SPD304, T8 and T23 in TNF and RANKL complexes.

Compound	TNF Complexes				RANKL Complexes		
	L929 IC_50_ (μM)	L929 LC_50_ (μM)	ELISA IC_50_ (μM)	K_d_ (μM)	IC_50_ (μM)	LC_50_ (μM)	K_d_ (μM)
**SPD304**[62]	5±0.2	7.5±0.2	5±0.2	5.8±0.5	0.9±0.1	3.2±0.1	13.8±0.7
**T8**	40±2.3	>100	30±2.3	8.8±0.8	9.0±0.7	42.6±4.3	6.3±0.6
**T23**	17±1.2	>100	3±0.1	2.8±0.2	2.6± 0.2	5.9±0.3	7.3±0.4

The two proposed molecular scaffolds were also explored in a structure-based scheme to further investigate binding to TNF and RANKL. For this purpose, we used the jFATCAT pairwise structure alignment algorithm[64] to align the RANKL structure (PDB code: 1S55) to the crystal structure of TNF dimer with SPD304 (PDB code: 2AZ5). We then performed molecular dynamics (MD) simulations and binding free energy calculations (MM–PBSA) for the SPD304, T8 and T23 complexes with TNF and RANKL to rationalize the docking calculations in a more rigorous fashion and to complement the experimental results with additional information. The MD analysis indicated the high stability of the complexes, since it was shown that the simulations eventually converged for all protein structures (S12 Fig). Indeed, Cα-based RMSD values for TNF in its complexes with the compounds were particularly stable, with slight fluctuations around an average deviation of approximately 2 Å from the crystal structure, while RANKL deviated more from the conformation of the crystal structure (~3.5 Å); however, it stabilized its structure after 35 ns in all complexes. The flexibility of individual TNF and RANKL residues in each complex is presented in S13 Fig. In general, RANKL residues appear more flexible than TNF residues and the highly flexible RANKL region around terminal residues 155–160 of monomer A in the T8 complex may suggest a less stable structure for the complex (S13 Fig). Additional information on radii of gyration and B-factors for the complexes is shown in S3 Table (Supporting Information).

**Fig 5:**
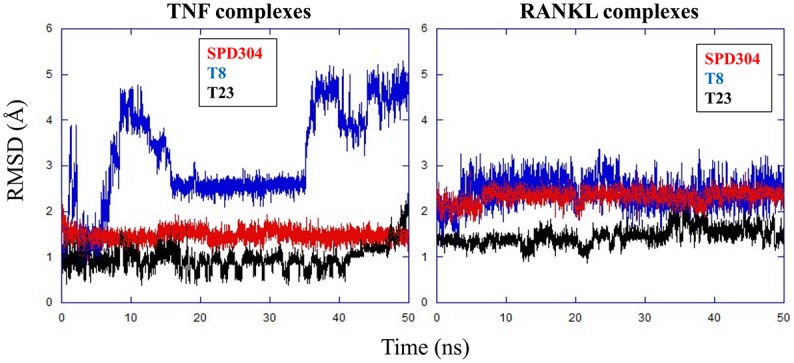
All-atom RMSD for compounds SPD304, T8 and T23 in complexes with TNF and RANKL.

All-atom RMSD calculations for each compound in either TNF or RANKL revealed differences in the binding modes of the complexes. As expected, SPD304 appears particularly stable in the binding sites of both proteins throughout the simulations (Fig 5). Interestingly, T23 also has very low RMSD values in its complexes (avg. ~1Å), thus indicating a high degree of stability. This may be due to enhanced interactions between T23 and the proteins, which suggest efficient T23 binding to TNF and RANKL. On the contrary, T8 displayed significant conformational changes throughout the runs, and especially in complex with TNF. In agreement with the experimental results, the increased flexibility of T8 in TNF may have reduced its binding to the protein.

The hydrogen bond (HB) analysis supported the experimental observations regarding the hydrophobic nature of the interactions that govern SPD304 binding to either protein target. It was observed that SPD304 was not involved in any HBs with TNF, thus further supporting the hypothesis of favorable hydrophobic interactions as the main driving force of SPD304–TNF binding. Similarly, HB interactions were not observed between SDP304 and RANKL.

On the other hand, it was suggested that despite the hydrophobic nature of the TNF and RANKL binding pockets, particular groups on T23 and T8 facilitate the formation of HBs with cavity residues. Therefore, the high stability of T23 in TNF and RANKL may be also attributed to HB networks between the ligand and particular TNF [Tyr119 (A chain), Ser60 (B chain), Gly121 (B chain)] and RANKL [Tyr214 (A chain), Trp192 (B chain), Ala193 (B chain)] residues. Also, the HB analysis on the T8 complexes may justify the significant structural variation of T8 in TNF compared to its relatively stable structure in RANKL, as depicted in Fig 5. Similar to SPD304, T8 did not participate in any HB interactions with TNF, and this may have enabled the molecule to frequently change conformations, whereas its structure is obviously restricted when bound to RANKL (Fig 5), mainly because it forms stable HBs with binding site residues [Asn275 (A chain), Gly277 (A chain), Gly278 (A chain), Asn275 (B chain)]. Representative conformations with main HBs between T8/T23 and TNF/RANKL (as obtained from the respective MD trajectories) are shown in S14 Fig.

**Fig 6:**
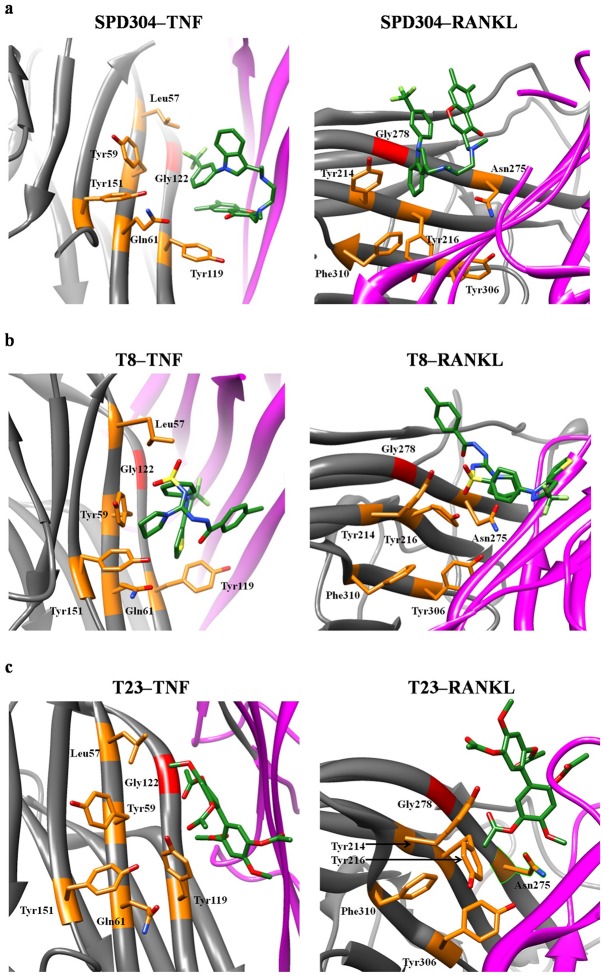
Representative MD conformations of (a) SPD304, (b) T8 and (c) T23 in TNF and RANKL complexes. Important residues for protein inhibition are displayed in orange; Gly122 and Gly278 are shown in red. Protein chains A and B are colored gray and magenta, respectively. For simplicity, interacting residues only on chains A are displayed and hydrogen atoms are not shown. Compounds are highlighted in green.

In agreement with the docking results, the compounds are in close proximity to the Leu120–Gly121–Gly122 *β* strand of TNF. This is particularly important, since it has been reported that SPD304 blocks the TNF trimerization and thus diminishes the biological activity of the protein by interacting with Gly122.[42] Additionally, the significance of Gly278 in RANKL trimer association has been indicated in several studies;[42,93,94] Douni *et al*.[42] have described an osteopetrotic mouse model that is based on a missense point mutation introducing a G278R substitution at the interface between the RANKL monomers. The mutated RANKL protein fails to form bioactive trimers, activate the RANK receptor, or induce osteoclastogenesis.[42] Modeling studies on SPD304–RANKL suggested that SPD304 should be located at an optimal position near Gly278 (~4 Å) to inhibit RANKL trimerization.[27] This observation coincides with the SPD304–Gly278 distance as observed from the 50 ns MD trajectory of the RANKL complex. Indeed, the average distance between the closest SPD304 atom and the Cα atom of Gly278 was calculated to be 4.25 ± 0.30 Å. Representative MD snapshots for the TNF and RANKL complexes with SPD304, T8 and T23 are presented in Fig 6. Residues Gly122 (TNF complexes) and Gly278 (RANKL complexes) are shown in red, while other residues (highlighted orange), such as Leu57, Gln61, Tyr59/119/151 (TNF complexes) and Tyr214/216/306, Asn275, Phe310 (RANKL complexes) are also in close proximity to the ligands and are considered crucial for either TNF or RANKL inhibition. For instance, TNF residue Tyr119 was observed to form stable HBs with T23, while RANKL residues (Asn275, Tyr214) show HB interactions with T8 and T23 as described above; other binding site amino acids, such as Tyr59/151, Gln61 and Leu57 have been reported as “contact residues” to SPD304 in the TNF crystal structure.[27]

MM–PBSA free energy calculations were carried out for each complex in order to validate the predictions of our models. Indeed, this analysis verified the experimental results, since it demonstrated that T8 and T23 have comparable binding energies to SPD304 (Table 5). Particularly, T8 and T23 binding energy values in TNF are close to the binding energy of SPD304, and they differ from each other by ~ 5 kcal/mol. It is important to highlight that such energy differences lie within the expected error of the method, and the corresponding binding energies are practically indistinguishable.[83,95] The high binding affinity of SPD304 to TNF in the absence of any HB interactions further supports the above claims regarding the hydrophobic nature of TNF binding. It is however predicted that T8 binding to TNF is associated with a high entropy penalty compared with the other compounds and also with its binding to RANKL (Table 5). This difference denotes the reduced hydrophobic character of the T8–TNF interaction and may also rationalize the above structural observation regarding the high conformational flexibility of T8 in TNF (Fig 5). On the other hand, the HB interaction between T8 and Asn275 (S14 Fig) in the RANKL complex may have resulted in a stable structure that is accompanied by a more favorable entropy term (Table 5). T23 appears to bind both protein targets equally strongly, with combined binding energy values that suggest most effective dual inhibition of TNF and RANKL than T8. Interestingly, in agreement with the potency experiments, our predictions ranked the three compounds with decreasing binding affinity to TNF as follows: SPD304 > T23 > T8. Finally, the MM–PBSA analysis revealed that the most favorable contributions to the binding enthalpy in all complexes are attributed to van der Waals interactions, followed by the nonpolar contribution to solvation, while the total electrostatics disfavor either TNF or RANKL complex formation.

**Table 5:** Energetic analysis for TNF and RANKL complexes with compounds SPD304, T8 and T23, as obtained by MM–PBSA calculations.

	TNF			RANKL		
Energy (kcal mol^-1^)	SPD304	T8	T23	SPD304	T8	T23
Δ*E*_vdW_	–36.96±0.17^a^	–36.84±0.08	–34.88±0.16	–25.92±0.15	–26.72±0.06	–35.20±0.08
Δ*E*_elec_	–0.03±0.02	–7.74±0.06	–9.01±0.07	–1.80±0.02	–10.45±0.10	–9.62±0.08
Δ*E*_MM, gas_	–36.99±0.18	–44.57±0.13	–43.89±0.20	–27.72±0.16	–37.17±0.12	–44.83±0.12
Δ*G*_PB_	13.60±0.07	22.39±0.09	25.57±0.13	10.51±0.08	24.14±0.11	24.20±0.11
Δ*G*_elec(tot)_	13.57±0.07	14.65±0.10	16.56±0.14	8.71±0.08	13.69±0.15	14.58±0.14
Δ*G*_NP_	–2.70±0.01	–3.17±0.01	–2.67±0.01	–1.75±0.01	–1.91±0.01	–3.03±0.01
Δ*G*_solv_	10.90±0.06	19.22±0.09	22.90±0.12	8.76±0.07	22.23±0.11	24.20±0.11
**Δ*H***_**(MM+solv)**_	**–26.09±0.13**	**–25.35±0.06**	**–20.99±0.10**	**–18.96±0.10**	**–14.94±0.05**	**–20.63±0.08**
**Δ*S***	**17.03±2.02**	**21.97±1.23**	**15.30±2.01**	**13.55±1.96**	**12.79±2.01**	**14.34±2.00**
**Δ*G***	**–9.06±2.02**	**–3.38±1.23**	**–5.69±2.02**	**–5.41±1.97**	**–2.15±2.01**	**–6.29±2.00**

^*a*^Standard error of the mean.

**Fig 7:**
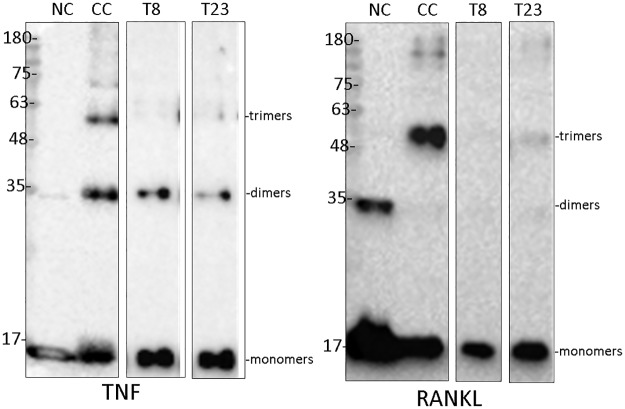
T8 and T23 obstruct the formation of active TNF and RANKL trimers. Human TNF or RANKL was incubated with T8 or T23, chemically cross-linked, and subjected to SDS-PAGE. This was followed by western blotting to detect the various TNF and RANKL multimers. Both inhibitors were used at a molar ratio of 4:1 relative to TNF and 1:1 relative to RANKL. Numbers indicate molecular weights in kDa; NC = non cross-linked control (no inhibitor, no cross-linking); CC = cross-linked control (no inhibitor).

As the modeled mechanism of action of T8 and T23 on both TNF and RANKL consists of an obstruction to the trimerization interface, the effect of inhibition at the level of trimerization was examined using chemical cross-linking experiments. As can be seen in Fig 7, both inhibitors can prevent the formation of trimers both for TNF and RANKL, when used at specific molar ratios. These results confirm that not only T8 and T23 are direct inhibitors of TNF and RANKL but also they do so by affecting the biologically-active configuration of these molecules, i.e. the trimeric form.

Finally, we addressed aggregation-induced and general non-specific effects in two ways. Firstly, by plotting saturation curves using the binding assay raw data and performing Scatchard analysis. For all protein/ligands tested (TNF/T8, TNF/T23, RANKL/T8 and RANKL/T23), the linearity of the Scatchard plots resulted in an ‘n’ value of 1, suggesting that there is only one ligand binding site per protein molecule. Representative binding and saturation curves and Scatchard plots are illustrated in S15 and S16 Figs. Secondly, we did not find evidence for non-specific effects due to aggregation as judged by the method proposed by Feng & Stoichet [96] (Table 6). Briefly, we examined the inhibitory effect of T8 and T23, as well as SPD304, on TNF and RANKL, using varying concentrations of the two tested proteins in the presence of varying concentrations of solubility enhancing agent PEG3350. Inhibition remained practically unchanged by fluctuating these two concentration parameters, indicating that both small molecules inhibit TNF and RANKL via specific mechanisms as proposed by our computational model.

**Table 6:** Physicochemical properties of SPD304, T8 and T23, and determination of their dissociation constant with TNF or RANKL under different assay conditions. PEG3350 was used as co-solvent in order to enhance inhibitors solubility and thus eliminate the possibility of aggregates formation.

Compound	clogP^a^			Binding affinity^c^ (K_d_, μM)^d^					
				TNF^e^ (μM)			RANKL^f^ (μM)		
		PEG3350 (% v/v)	Solubility (μM)	0.75	1.5	2.5	0.5	1.5	2.5
SPD-304	8.00	0	10^b^	N.T.	N.T.	N.T.	N.T.	N.T.	N.T.
		2.5	71	6.18 ± 0.54	N.T.	N.T.	14.21 ± 0.68	N.T.	N.T.
		5	84^b^	5.76 ± 0.54	5.45 ± 0.43	5.61 ± 0.22	13.82 ± 0.73	14.08. ± 0.77	13.58. ± 0.63
T8	6.41	0	<1	N.T.	N.T	N.T	N.T	N.T.	N.T.
		2.5	32	9.55 ± 0.81	N.T.	N.T.	6.15 ± 0.22	N.T.	N.T.
		5	48	8.78 ± 0.76	9.02 ± 0.88	9.17 ± 0.88	6.32 ± 0.62	6.55 ± 0.84	6.05 ± 0.37
T23	-0.07	0	36	2.53 ± 0.23	N.T.	N.T.	7.02 ± 0.63	N.T.	N.T.
		2.5	88	3.02 ± 0.31	N.T.	N.T	7.44 ± 0.52	N.T.	N.T.
		5	117	2.80 ± 0.19	2.63 ± 0.25	2.98 ± 0.47	7.25 ± 0.44	7.62 ± 0.84	7.02 ± 0.33

^a^Calculated using ChemDraw

^b^Data obtained from [85]

^c^Determination of binding affinity to TNF or RANKL by fluorescence assay

^d^Mean ± SE (n = 3 independent experiments); *p* < 0.05

^e^Experiments were performed in the presence of different concentrations of TNF in 10 mM Citrate-phosphate buffer (pH 6.5) containing either 0 or 2.5 or 5% PEG3350

^f^Experiments were performed in the presence of different concentrations of RANKL in 25 mM Tri-HCl buffer, 100 mM NaCl (pH 7.5) containing either 0 or 2.5 or 5% PEG3350

N.T.: not tested

Collectively, our computational approach succeeded to identify two compounds that were experimentally evaluated as dual TNF and RANKL inhibitors, with T8 being the one with the lowest toxicity. Within the proposed strategy, the Enalos Cloud Platform emerges as a key component for the evaluation of novel small molecules that have not been experimentally evaluated or even synthesized and can also be expanded for several drug-related properties (e.g., inclusion of toxicity and solubility models). Our proposed methodology and tools can also be expanded and applied to other biological targets that are now gaining attention. Specifically, in the context of developing novel treatments for chronic inflammatory diseases, the proposed molecular scaffolds, T8 and T23, could be investigated as lead compounds for future drug design targeting TNF and RANKL inhibition.

### Conclusions

In this work, we have identified computationally and validated experimentally two small-molecule compounds that function as direct inhibitors of TNF by blocking the protein–protein interaction (PPI) between the cytokine and its receptor. Both compounds (T8 and T23) were confirmed to be direct inhibitors of TNF function, with IC_50_ values comparable to those of the potent inhibitor SPD304, and showed low toxicity even at concentrations above 100 μM. Our computational approach combined structure–based modeling with ligand-based modeling. The predictive ligand-based model was made publicly available[60] through the Enalos Cloud Platform (http://enalos.insilicotox.com/TNFPubChem) and can be used for the predictions of TNF inhibition (specific NF-κB induction) of novel small molecules.

Most importantly, our proposed small molecules, T8 and T23, were validated experimentally to act as dual inhibitors, being able to hinder both TNF and RANKL function at the low micromolar range; thus, they are proposed as the second and third examples of dual PPI inhibitors of TNF and RANKL. T8 and T23 were proven to directly bind to both TNF and RANKL and affect the formation of biologically-active trimers as predicted by our computation model. Furthermore, molecular dynamics calculations provided complementary information regarding the interactions at the molecular level of the two compounds in the TNF/RANKL complexes. The proposed molecular scaffolds could be further optimized in drug design targeting TNF and RANKL, ultimately aiming at the development of novel treatments for a range of inflammatory and autoimmune diseases.

## Supporting information

S1 Fig Inhibition of TNF-induced death in L929 cells. (TIF)

S2 Fig Toxicity of T8 and T23 inhibitors. (TIF)

S3 Fig Structure and NMR spectra of T23. (TIF)

S4 Fig Structure and NMR spectra of T8. (TIF)

S5 Fig MS spectrum of T8. (TIF)

S6 Fig MS spectrum of T23. (TIF)

S7 Fig Disruption of the TNF/TNFR1 interaction. (TIF)

S8 Fig Dissociation constant (K_d_) determination of TNF with SPD304 (A1, A2), T8 (B1, B2) and T23 (C1, C2) at 25°C in 10 mM Citrate-Phosphate pH 6.2 containing 5% PEG3350; λ_ex_ = 274 nm/λ_em_ = 302 nm. (TIF)

S9 Fig Effects of T8 and T23 on RANKL-induced osteoclast activity. (TIF)

S10 Fig Toxicity effects of T8 and T23 on primary BM cells. (TIF)

S11 Fig Dissociation constant (K_d_) determination of RANKL with SPD304 (A1, A2), T8 (B1, B2) and T23 (C1, C2) at 25°C in 25 mM Tris-HCl, 100 mM NaCl, pH 7.5 containing 5% PEG3350; λ_ex_ = 274 nm/λ_em_ = 302 nm. (TIF)

S12 Fig RMSD for Cα atoms of TNF and RANKL residues in their complexes with SPD304, T8 and T23. (TIF)

S13 Fig Cα atomic fluctuations for TNF and RANKL residues in complexes with SPD304, T8 and T23. (TIF)

S14 Fig Representative MD conformations of (a) T8 and (b) T23 in TNF and RANKL complexes. (TIF)

S15 Fig Examination of non-specific effects on the TNF/T8 interaction. Changes of TNF fluorescence (λ_ex_ = 274 nm/λ_em_ = 302 nm) were measured after incubation of TNF with T8 at 25°C (A). Saturation plots after calculation of free (L) and bound (PL) concentrations when measurements were obtained in the presence of 2.5% (A.1) and 5% (A.2) PEG3350, respectively. Insets: Scatchard plots. The mean values of three independent measurements are presented.
(TIF)

S16 Fig Examination of non-specific effects on the TNF/T23 interaction. Changes of TNF fluorescence (λ_ex_ = 274 nm/λ_em_ = 302 nm) were measured after incubation of TNF with T23 at 25°C (A). Saturation plots after calculation of free (L) and bound (PL) concentrations when measurements were obtained in the absence of PEG3350 (A.1) or in the presence of 2.5% (A.2) and 5% (A.3) PEG3350, respectively. Insets: Scatchard plots. The mean values of three independent measurements are presented.
(TIF)

S1 Table Structure–based predictions. Ranks of identified hits.
(DOCX)

S2 Table Mold2 descriptors used in this study. (DOCX)

S3 Table B–factors and radii of gyration for TNF and RANKL complexes. (DOCX)

S1 Text Methods. (DOCX)

S2 Text Results. (DOCX)
