# Supplementary figures and images for: Cheminformatics-aided discovery of small-molecule Protein-Protein Interaction (PPI) dual inhibitors of Tumor Necrosis Factor (TNF) and Receptor Activator of NF-κB Ligand (RANKL)
Source: PLoS Comput Biol. 2017 Apr 20;13(4):e1005372. doi: 10.1371/journal.pcbi.1005372 (PMC5398486; doi:10.1371/journal.pcbi.1005372)

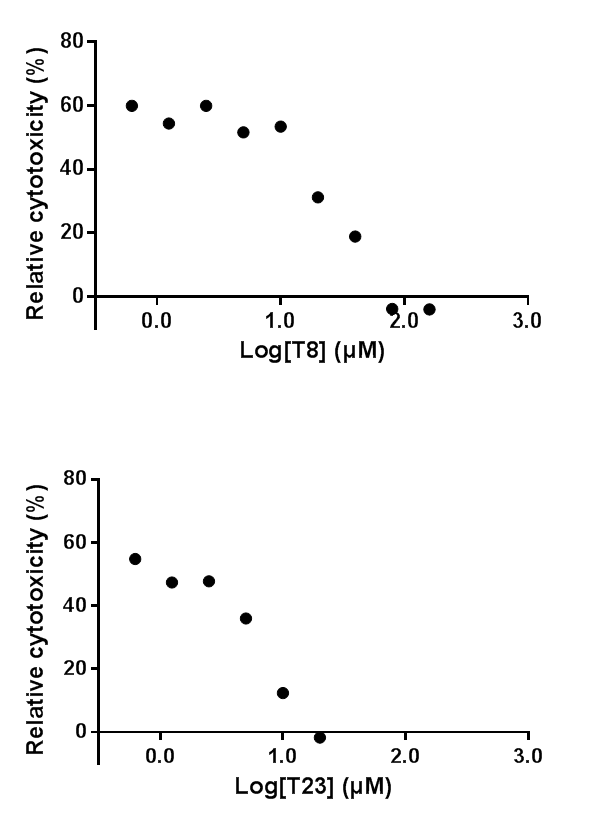

Supplement: S1 Fig — (TIF) [file pcbi.1005372.s001.tif]

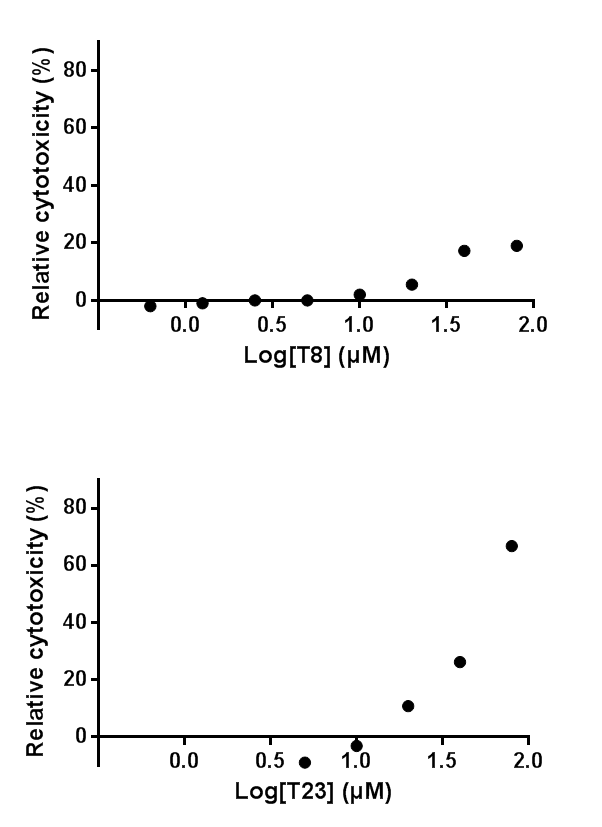

Supplement: S2 Fig — (TIF) [file pcbi.1005372.s002.tif]

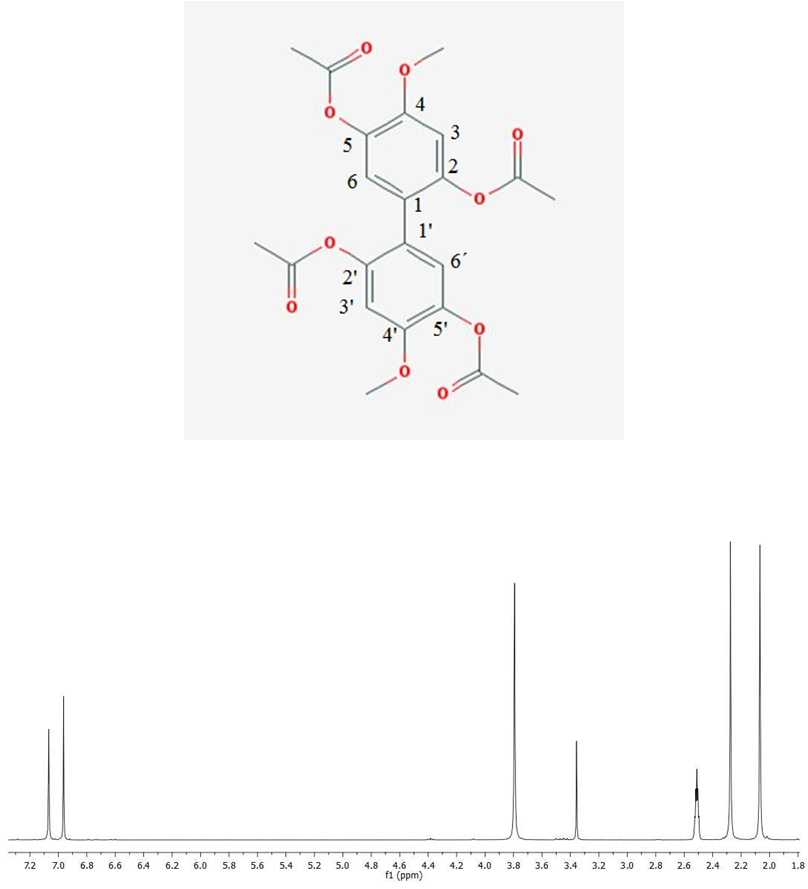

Supplement: S3 Fig — (TIF) [file pcbi.1005372.s003.tif]

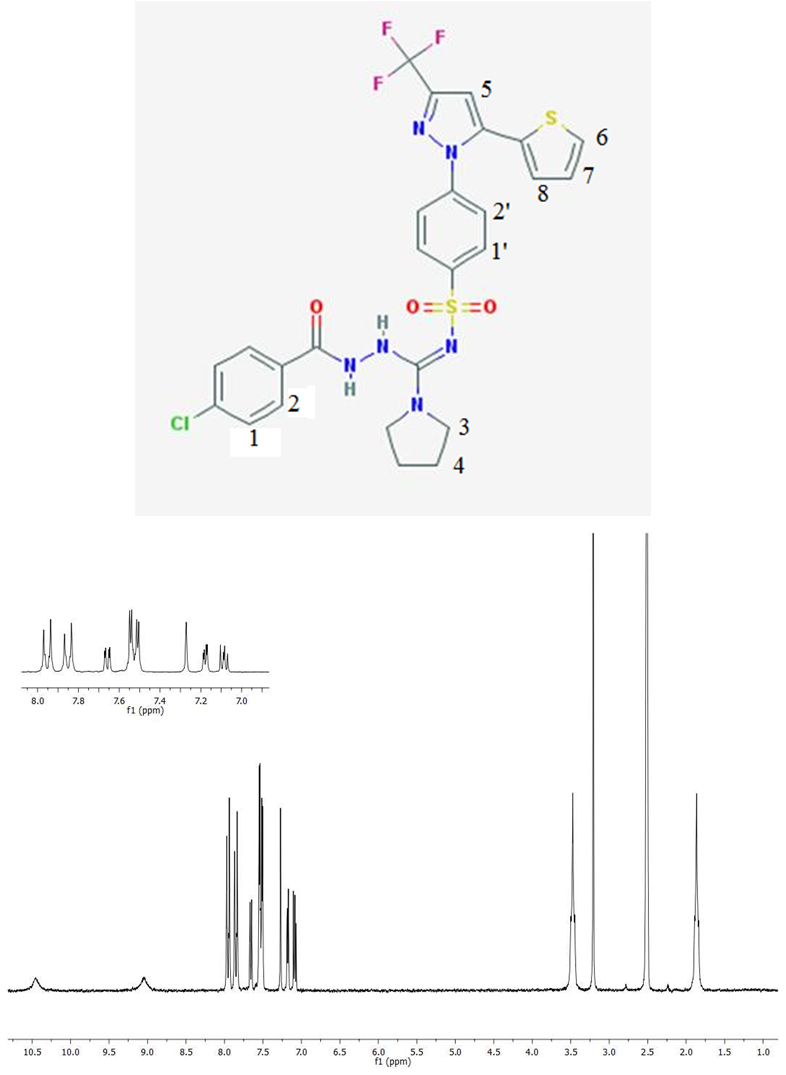

Supplement: S4 Fig — (TIF) [file pcbi.1005372.s004.tif]

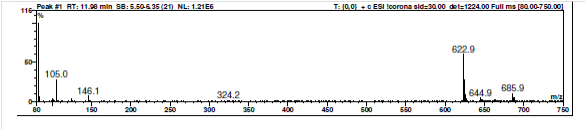

Supplement: S5 Fig — (TIF) [file pcbi.1005372.s005.tif]

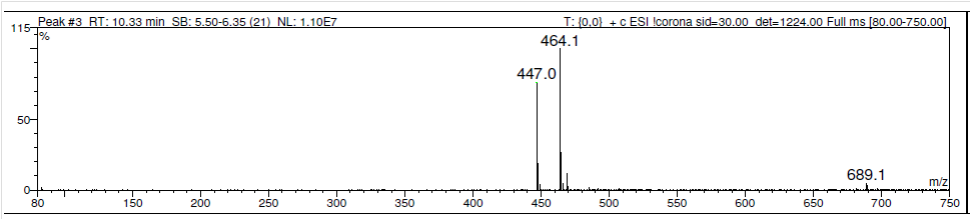

Supplement: S6 Fig — (TIF) [file pcbi.1005372.s006.tif]

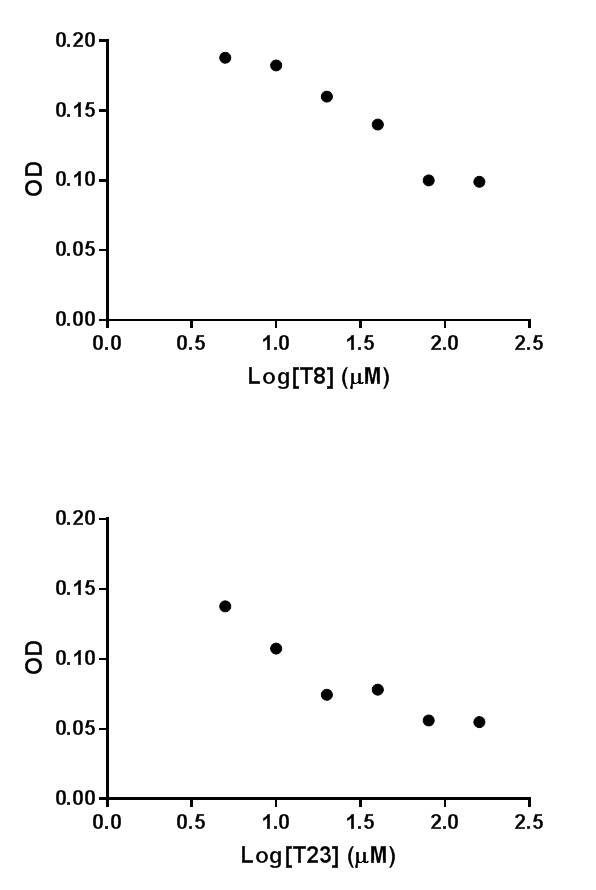

Supplement: S7 Fig — (TIF) [file pcbi.1005372.s007.tif]

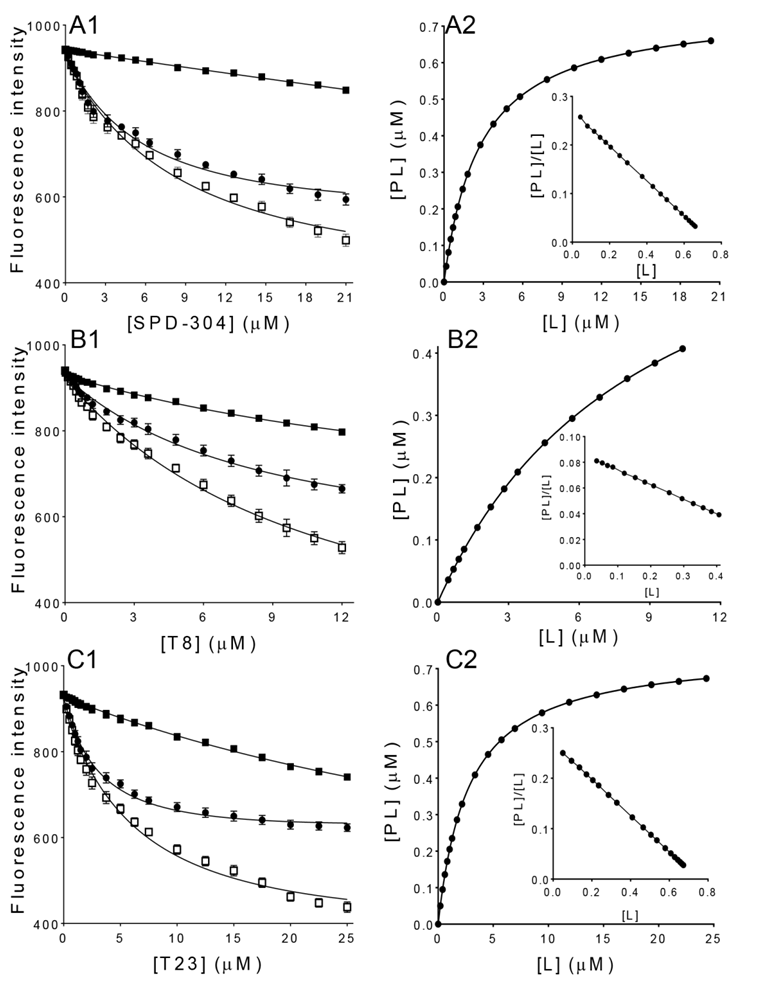

Supplement: S8 Fig — (TIF) [file pcbi.1005372.s008.tif]

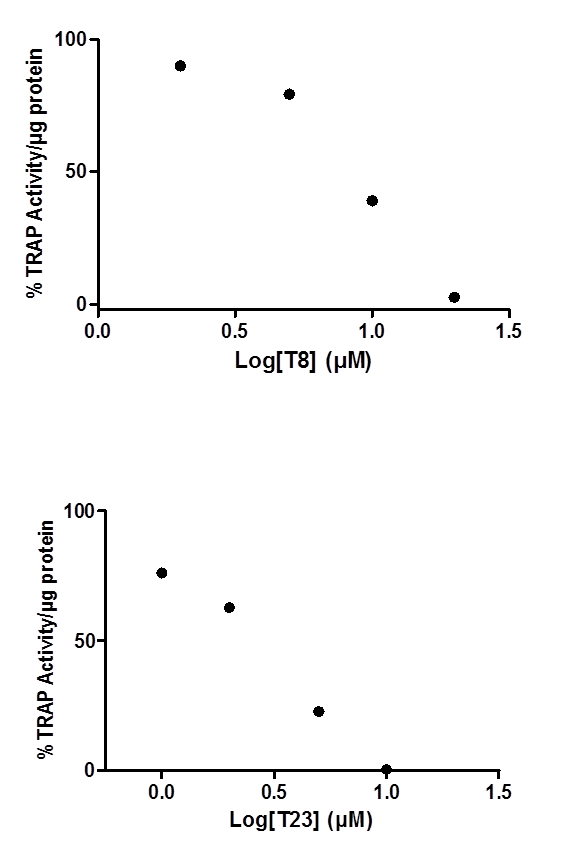

Supplement: S9 Fig — (TIF) [file pcbi.1005372.s009.tif]

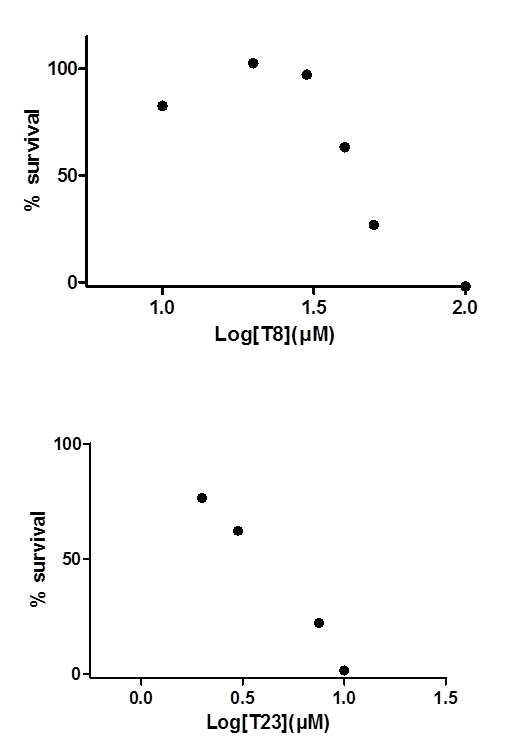

Supplement: S10 Fig — (TIF) [file pcbi.1005372.s010.tif]

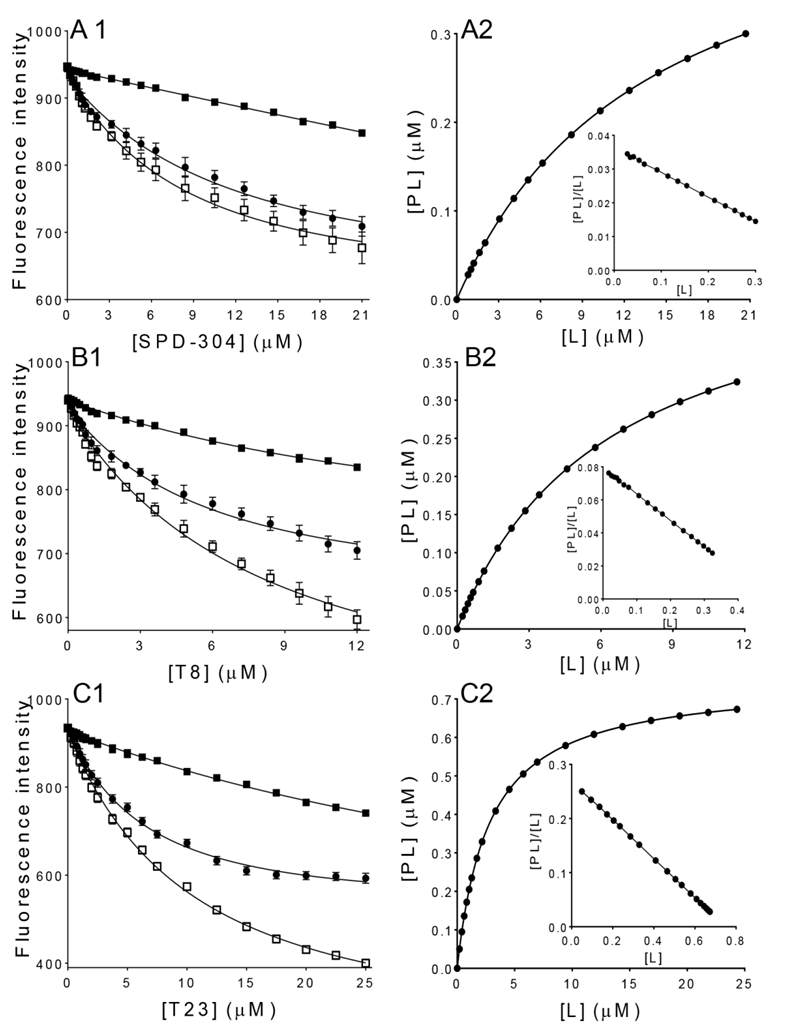

Supplement: S11 Fig — (TIF) [file pcbi.1005372.s011.tif]

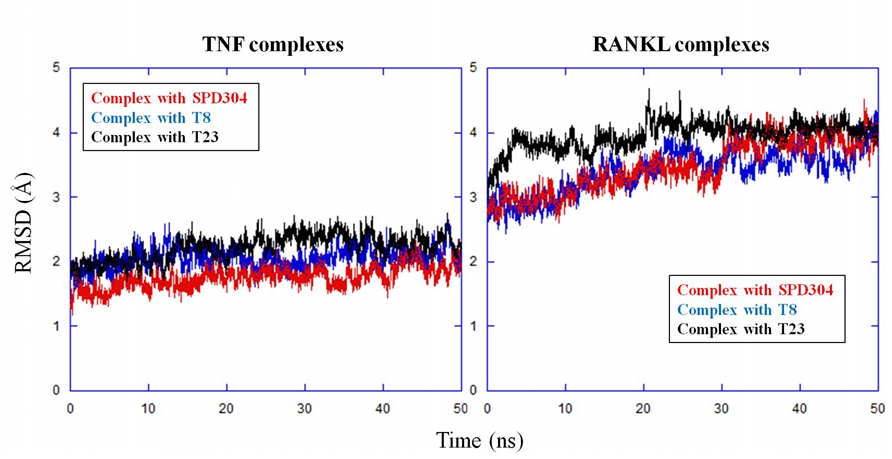

Supplement: S12 Fig — (TIF) [file pcbi.1005372.s012.tif]

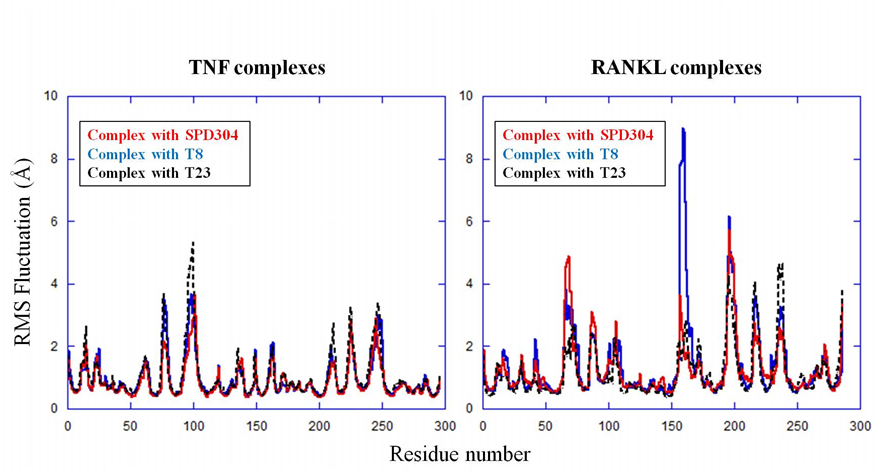

Supplement: S13 Fig — (TIF) [file pcbi.1005372.s013.tif]

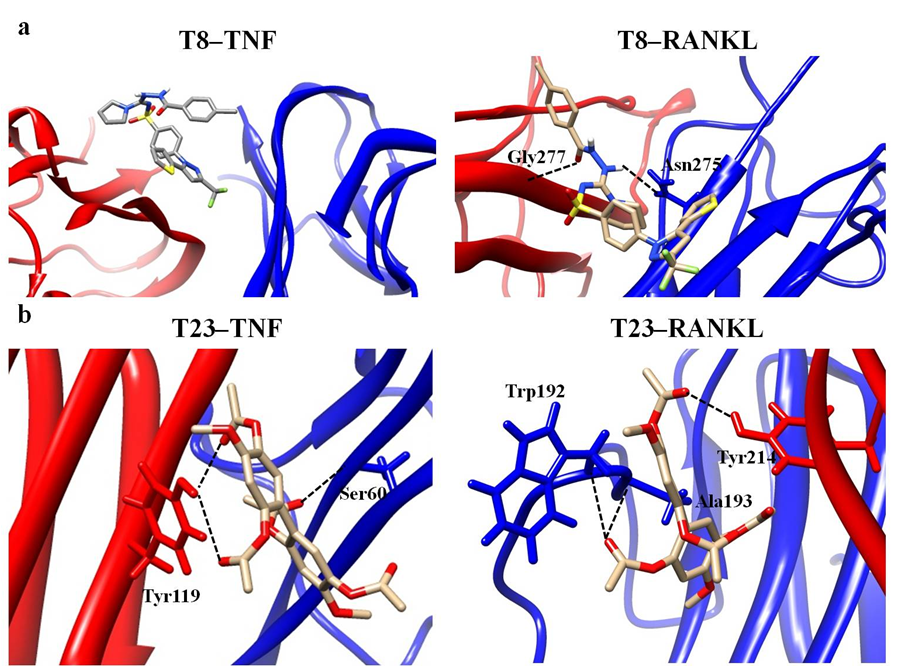

Supplement: S14 Fig — (TIF) [file pcbi.1005372.s014.tif]

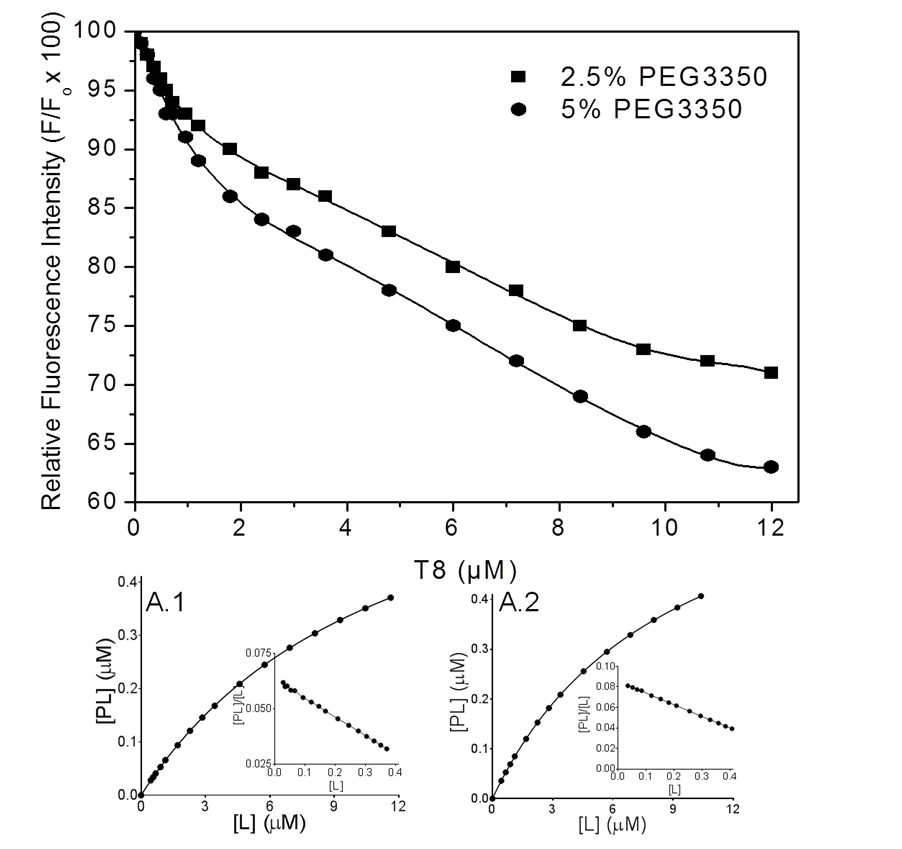

Supplement: S15 Fig — Changes of TNF fluorescence (λex = 274 nm/λem = 302 nm) were measured after incubation of TNF with T8 at 25°C (A). Saturation plots after calculation of free (L) and bound (PL) concentrations when measurements were obtained in the presence of 2.5% (A.1) and 5% (A.2) PEG3350, respectively. Insets: Scatchard plots. The mean values of three independent measurements are presented. (TIF) [file pcbi.1005372.s015.tif]

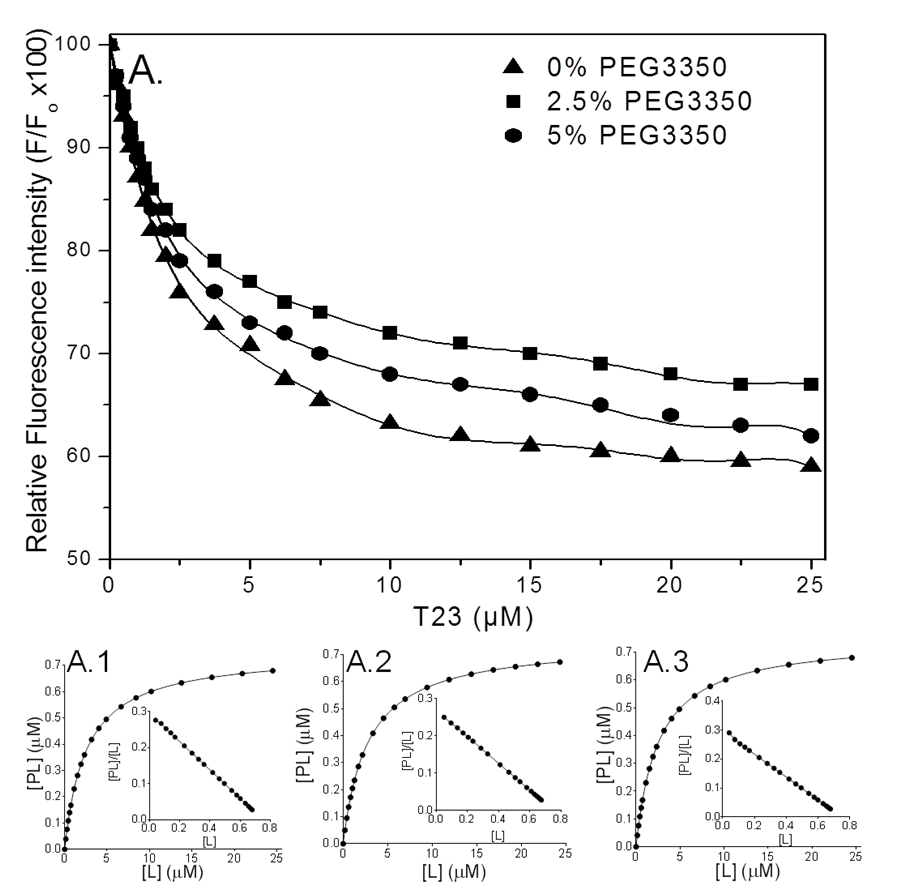

Supplement: S16 Fig — Changes of TNF fluorescence (λex = 274 nm/λem = 302 nm) were measured after incubation of TNF with T23 at 25°C (A). Saturation plots after calculation of free (L) and bound (PL) concentrations when measurements were obtained in the absence of PEG3350 (A.1) or in the presence of 2.5% (A.2) and 5% (A.3) PEG3350, respectively. Insets: Scatchard plots. The mean values of three independent measurements are presented. (TIF) [file pcbi.1005372.s016.tif]
