# Supplementary material for: Cheminformatics-aided discovery of small-molecule Protein-Protein Interaction (PPI) dual inhibitors of Tumor Necrosis Factor (TNF) and Receptor Activator of NF-κB Ligand (RANKL)
Source: PLoS Comput Biol. 2017 Apr 20;13(4):e1005372. doi: 10.1371/journal.pcbi.1005372 (PMC5398486; doi:10.1371/journal.pcbi.1005372)
Supplement: S1 Table — Ranks of identified hits. (DOCX) [file pcbi.1005372.s017.docx]

**S1 Table.** Structure–based predictions. Ranks of identified hits.

| ID | Docking Score  –logK_d_ | InChI |
| --- | --- | --- |
| 1 | 10.54 | InChI=1S/C28H33N3O4S/c32-26-21-28(18-17-22-9-3-1-4-10-22,27(33)31(26)24-11-5-2-6-12-24)29-23-13-15-25(16-14-23)36(34,35)30-19-7-8-20-30/h1,3-4,9-10,13-18,24,29H,2,5-8,11-12,19-21H2/b18-17+/t28-/m0/s1 |
| 2 | 9.78 | InChI=1S/C27H29NO4/c1-20(28-26(29)18-21-9-5-3-6-10-21)13-14-23-15-16-24(25(17-23)31-2)32-27(30)19-22-11-7-4-8-12-22/h3-12,15-17,20H,13-14,18-19H2,1-2H3,(H,28,29)/t20-/m1/s1 |
| 3 | 9.5 | InChI=1S/C25H15F3N2O6/c26-25(27,28)16-12-13-20(19(15-16)30(32)33)35-21-10-4-5-11-22(21)36-24(31)18-9-6-14-29-23(18)34-17-7-2-1-3-8-17/h1-15H |
| 4 | 9.42 | InChI=1S/C26H28FN3O4/c1-31-23-9-8-17(13-24(23)32-2)12-22-21-16-26(34-4)25(33-3)14-18(21)10-11-30(22)29-28-20-7-5-6-19(27)15-20/h5-9,13-16,22H,10-12H2,1-4H3/b29-28+/t22-/m0/s1 |
| 5 | 9.27 | InChI=1S/C21H19Cl3N4O3/c1-12-20(30-18-5-3-15(22)4-6-18)13(2)28(26-12)8-7-19(25)27-31-21(29)14-9-16(23)11-17(24)10-14/h3-6,9-11H,7-8H2,1-2H3,(H2,25,27)/p+1 |
| 6 | 9.14 | InChI=1S/C28H35NO8S2/c1-6-35-28(36-7-2)20-29(38(30,31)24-13-8-21(3)9-14-24)19-23-12-17-26(34-5)27(18-23)37-39(32,33)25-15-10-22(4)11-16-25/h8-18,28H,6-7,19-20H2,1-5H3 |
| 7 | 9 | InChI=1S/C19H25N5O4/c1-13(25)21-14-6-4-7-15(10-14)28-9-5-8-24-11-16-17(20-12-24)22(2)19(27)23(3)18(16)26/h4,6-7,10,20H,5,8-9,11-12H2,1-3H3,(H,21,25)/p+1 |
| 8 | 8.86 | InChI=1S/C22H17BrO4/c1-25-18-11-10-15(23)12-14(18)13-26-22(24)21-16-6-2-4-8-19(16)27-20-9-5-3-7-17(20)21/h2-12,21H,13H2,1H3 |
| 9 | 8.86 | InChI=1S/C25H27NO2/c1-19-10-13-24-21(15-19)9-6-14-26(24)17-22-11-12-23(16-25(22)27-2)28-18-20-7-4-3-5-8-20/h3-5,7-8,10-13,15-16H,6,9,14,17-18H2,1-2H3 |
| 10 | 8.72 | InChI=1S/C20H29NO12/c1-11(22)29-10-16(30-12(2)23)17(31-13(3)24)18(32-14(4)25)19(33-15(5)26)20(27)21-6-8-28-9-7-21/h16-19H,6-10H2,1-5H3/t16-,17-,18+,19-/m1/s1 |
| 11 | 8.71 | InChI=1S/C21H25N3O9S/c1-4-30-21(25)14-12-17(31-10-8-28-2)18(32-11-9-29-3)13-16(14)24-34(26,27)19-7-5-6-15-20(19)23-33-22-15/h5-7,12-13,24H,4,8-11H2,1-3H3 |
| 12 | 8.65 | InChI=1S/C28H26ClN3O2/c1-18-26(27(31-34-18)22-12-6-7-13-23(22)29)28(33)30-24-14-8-4-10-20(24)17-25-21-11-5-3-9-19(21)15-16-32(25)2/h3-14,25H,15-17H2,1-2H3,(H,30,33)/t25-/m0/s1 |
| 13 (T8) | 8.65 | InChI=1S/C26H22ClF3N6O3S2/c27-18-7-5-17(6-8-18)24(37)31-32-25(35-13-1-2-14-35)34-41(38,39)20-11-9-19(10-12-20)36-21(22-4-3-15-40-22)16-23(33-36)26(28,29)30/h3-12,15-16,25,34H,1-2,13-14H2/b32-31+/t25-/m0/s1 |
| 14 | 8.62 | InChI=1S/C20H20N2O5S3/c23-29(24,16-6-2-1-3-7-16)19-10-11-20(28-19)30(25,26)21-17-8-4-5-9-18(17)22-12-14-27-15-13-22/h1-11,21H,12-15H2 |
| 15 (T23) | 8.57 | InChI=1S/C22H22O10/c1-11(23)29-17-9-19(27-5)21(31-13(3)25)7-15(17)16-8-22(32-14(4)26)20(28-6)10-18(16)30-12(2)24/h7-10H,1-6H3 |
| 16 | 8.54 | InChI=1S/C27H19ClN8/c28-21-13-11-19(12-14-21)25-20(17-35(34-25)22-7-3-1-4-8-22)15-31-33-26-24-16-32-36(27(24)30-18-29-26)23-9-5-2-6-10-23/h1-14,16-18H,15H2/b33-31+ |
| 17 | 8.51 | InChI=1S/C27H26N2O5/c1-18(30)29-26(21-13-15-22(32-3)16-14-21)27(34-19(2)31)25(28-29)23-11-7-8-12-24(23)33-17-20-9-5-4-6-10-20/h4-16,26-27H,17H2,1-3H3/t26-,27-/m0/s1 |
| 18 | 8.49 | InChI=1S/C19H36N4OS/c1-3-4-5-6-7-8-9-10-11-12-13-14-15-20-18(24)16-25-19-22-21-17-23(19)2/h17H,3-16H2,1-2H3,(H,20,24) |
| 19 | 8.45 | InChI=1S/C22H23NO11/c1-10(24)30-9-16-18(31-11(2)25)19(32-12(3)26)17(22(34-16)33-13(4)27)23-20(28)14-7-5-6-8-15(14)21(23)29/h5-8,16-19,22H,9H2,1-4H3/t16-,17-,18-,19-,22-/m1/s1 |
| 20 | 8.43 | InChI=1S/C25H26F3N3O2/c1-32-23-15-22(33-18-19-5-3-2-4-6-19)9-7-20(23)17-30-11-13-31(14-12-30)24-10-8-21(16-29-24)25(26,27)28/h2-10,15-16H,11-14,17-18H2,1H3 |
| 21 | 8.42 | InChI=1S/C20H21N3O4S/c1-14-17(20(22-27-14)15-6-4-3-5-7-15)13-21-28(24,25)16-8-9-19-18(12-16)23(2)10-11-26-19/h3-9,12,21H,10-11,13H2,1-2H3 |
| 22 | 8.41 | InChI=1S/C26H30N4O2S2/c1(3-11-17-23-27-29-25(31-23)33-19-21-13-7-5-8-14-21)2-4-12-18-24-28-30-26(32-24)34-20-22-15-9-6-10-16-22/h5-10,13-16H,1-4,11-12,17-20H2 |
| 23 | 8.38 | InChI=1S/C24H26N4O4S2/c1-17-7-9-19(10-8-17)34(31,32)28-13-11-27(12-14-28)16-22(29)26-20-15-21(33-23(20)24(25)30)18-5-3-2-4-6-18/h2-10,15H,11-14,16H2,1H3,(H2,25,30)(H,26,29) |
| 24 | 8.38 | InChI=1S/C23H18N6OS/c1-16-22(28-12-6-5-11-20(28)25-16)23(30)26-24-14-17-15-29(18-8-3-2-4-9-18)27-21(17)19-10-7-13-31-19/h2-15H,1H3,(H,26,30)/b24-14- |
| 25 | 8.3 | InChI=1S/C22H27NO11/c1-12(24)30-11-18(31-13(2)25)19(32-14(3)26)20(33-15(4)27)21(34-16(5)28)22(29)23-17-9-7-6-8-10-17/h6-10,18-21H,11H2,1-5H3,(H,23,29)/t18-,19-,20+,21-/m0/s1 |
| 26 | 8.26 | InChI=1S/C18H20FNO4S/c1-23-17-11-8-14(12-18(17)24-15-4-2-3-5-15)20-25(21,22)16-9-6-13(19)7-10-16/h6-12,15,20H,2-5H2,1H3 |
| 27 | 8.25 | InChI=1S/C25H27NO2S/c1-28-24-12-5-4-11-23(24)20-13-15-26(16-14-20)25(27)18-29-17-21-9-6-8-19-7-2-3-10-22(19)21/h2-12,20H,13-18H2,1H3 |
| 28 | 8.22 | InChI=1S/C17H16N4O/c22-17(11-21-16-8-4-3-7-15(16)19-20-21)18-14-9-12-5-1-2-6-13(12)10-14/h1-8,14H,9-11H2,(H,18,22) |
| 29 | 8.21 | InChI=1S/C25H32N2O2/c1-3-29-25(28)20-12-15-26(16-13-20)23-9-5-4-7-22(23)18-27-14-6-8-21-17-19(2)10-11-24(21)27/h4-5,7,9-11,17,20H,3,6,8,12-16,18H2,1-2H3 |
| 30 | 8.11 | InChI=1S/C17H21N5O3S2/c23-27(24,15-8-2-1-3-9-15)21-16(22-10-4-5-11-22)19-20-17(26)18-13-14-7-6-12-25-14/h1-3,6-9,12,16,21H,4-5,10-11,13H2,(H,18,26)/b20-19+/t16-/m0/s1 |
