# Supplementary material for: Cheminformatics-aided discovery of small-molecule Protein-Protein Interaction (PPI) dual inhibitors of Tumor Necrosis Factor (TNF) and Receptor Activator of NF-κB Ligand (RANKL)
Source: PLoS Comput Biol. 2017 Apr 20;13(4):e1005372. doi: 10.1371/journal.pcbi.1005372 (PMC5398486; doi:10.1371/journal.pcbi.1005372)
Supplement: S2 Table — (DOCX) [file pcbi.1005372.s018.docx]

**S2 Table**. Mold2 descriptors used in this study.

| **Descriptor** | **Feature selection method** | **Description** |
| --- | --- | --- |
| *D263* | I,II | The vertex distance path count index |
| *D314* | I,II | The sum eigenvalue weighted by electronegativity Allred-Rochow-Scale distance matrix^a^ |
| *D580* | I | The highest eigenvalue from Burdex matrix weighted by electronegativities Sanderson-Scale order-1^b^ |
| *D661* | I,II,III | The number of ammonium groups (aliphatic) in the molecule |
| *D662* | I,II,III | The number of ammonium groups (aromatic) in the molecule |
| *D687* | I,II,III | The number of thiol groups in the molecule |
| *D730* | I | The number of =CR_2_ groups in the molecule |
| *D747* | I,II | The number of H atoms attached to a heteroatom |
| *D712* | II | The number of donor atoms for H-bonds (with N and O) |
| *D487* | II | Moran topological structure autocorrelation length-1 weighted by atomic van der Waals volumes^c^ |
| *D274* | II | Information content order-5 index (neighborhood symmetry of 5-order) |
| *D488* | II | Moran topological structure autocorrelation length-2 weighted by atomic van der Waals volumes |
| *D769* | II,III | The number of R-SH groups |
| *D297* | II | Bond information content order-4 index (neighborhood symmetry of 4-order) |
| *D490* | II | Moran topological structure autocorrelation length-4 weighted by atomic van der Waals volumes |
| *D775* | II | The hydrophilic factor index^d^ |
| *D254* | II | The radial centric index^e^ |
| *D313* | III | The sum eigenvalue weighted by electronegativity Sanderson-Scale distance matrix |
| *D196* | III | The sum absolute electrotopological negative variation^f^ |
| *D417* | III | The topological structure autocorrelation length-3 weighted by atomic masses |
| *D310* | III | The sum eigenvalue weighted by van der Waals distance matrix |
| *D601* | III | The number of ring tertiary C-sp3 |
| *D377* | III | The sum of topological distance between the vertices O and F |
| *D130* | III | The number of halogen atoms in each molecule |
| *D713* | III | The number of group acceptor atoms for H-bonds (N, O, F) |
| *D715* | III | The number of CH_2_R_2_ groups |
| *D026* | III | The number of oxygen atoms that are present in the molecule |

Notes: ^a^The Allred-Rochow electronegativity approximates the electrostatic force exerted by the effective nuclear charge on the valence electrons. The values of the effective nuclear charges are derived from Slater's rules in a partly empirical way.

^b^Sanderson’s electronegativity equalization assumes that electron distribution around a molecule occurs in order to minimize (or equalize) the Mulliken electronegativity.

^c^This descriptor is defined as *I_d_ (P)* with d = 1,2,..,10. The form of function *I_d_ (P)* is:^86^ $I_{d}\left( P \right)=\frac{\frac{1}{\Delta}\sum_{i=1}^{N} \sum_{j=1}^{N} \delta_{ij}\left( P_{i}-\bar{P} \right)(P_{j}-\bar{P})}{\frac{1}{N}\sum_{i=1}^{N} {(P_{i}-\bar{P})}^{2}}$

where *P* and $\bar{P}$ are any atomic property and its average value, respectively; N is the number of atoms; *d* is the topological distance; and 𝛿_𝑖𝑗_ is the Kronecker delta. Δ defines the sum of 𝛿_𝑖𝑗_.

^d^This accounts for the hydrophilicity of each of the compounds described.

^e^This index is defined as:^87^ ${}^{V}{\bar{I}_{C,R}=-\sum_{g=1}^{G} \frac{n_{g}}{A}{log}_{2}\frac{n_{g}}{A}}$

where n_g_ is the number of graph vertices having the same maximum distance from a vertex to any other vertex in the graph (atom eccentricity); G is the number of different vertex equivalence classes; and A is the number of graph vertices.

^f^The electrotopological state index considers the electronic effect of each atom on the other atoms within a molecule. The E-state for each atom is defined as the modified intrinsic state value of the atom, after the perturbing effect of the other atoms. D196 provides the sum of the negative numbers in of the E-state index.
