# Supplementary material for: Cheminformatics-aided discovery of small-molecule Protein-Protein Interaction (PPI) dual inhibitors of Tumor Necrosis Factor (TNF) and Receptor Activator of NF-κB Ligand (RANKL)
Source: PLoS Comput Biol. 2017 Apr 20;13(4):e1005372. doi: 10.1371/journal.pcbi.1005372 (PMC5398486; doi:10.1371/journal.pcbi.1005372)
Supplement: S3 Table — (DOCX) [file pcbi.1005372.s019.docx]

**S3 Table.** B–factors and radii of gyration for TNF and RANKL complexes.

| Protein | Compound | B–factor (Å^2^)  average/median | Radius of gyration (Å)  average±STD deviation |
| --- | --- | --- | --- |
| TNF | SPD304 | 27.75/12.12 | 20.410±0.065 |
|  | T8 | 34.29/14.08 | 20.375±0.072 |
|  | T23 | 43.65/14.85 | 20.404±0.087 |
|  | SPD304 | 62.52/23.20 | 19.812±0.095 |
| RANKL | T8 | 93.53/21.27 | 19.778±0.105 |
|  | T23 | 53.62/18.13 | 19.531±0.107 |
