# Supplementary material for: Cheminformatics-aided discovery of small-molecule Protein-Protein Interaction (PPI) dual inhibitors of Tumor Necrosis Factor (TNF) and Receptor Activator of NF-κB Ligand (RANKL)
Source: PLoS Comput Biol. 2017 Apr 20;13(4):e1005372. doi: 10.1371/journal.pcbi.1005372 (PMC5398486; doi:10.1371/journal.pcbi.1005372)
Supplement: S1 Text — (DOCX) [file pcbi.1005372.s020.docx]

**Methods**

**Virtual screening using Surflex-Dock.** The Surflex-Dock algorithm was applied to treat compounds’ binding to TNF by allowing flexible structures for the ligands while the receptor was kept rigid (Jain, A. N. Surflex: Fully automatic flexible molecular docking using a molecular similarity-based search engine. *J. Med. Chem*. **2003**, *46*, 499−511). The molecular docking procedure involved the alignment of the ligand to a “protomol”, which is a molecular representation of an idealized ligand, in order to generate putative conformations of docked structures. The Hammerhead scoring function that includes hydrophobic, polar, repulsive, solvation and entropy terms has been applied. The protomol was constructed with the ligand-based mode, while parameters determining its extent, such as the threshold and bloat were kept at 0.50 and 0 Å, respectively. The docking procedure was initiated from randomly generated starting conformations per molecule; all other Surflex-Dock parameters assumed their default values. The docking scores are expressed in –logK_d_ (S1 Table).

**Ligand–based *in silico* model**

Validation metrics used to assess the predictive power of the ligand-based model:

Precision = $\frac{\text{TP}}{\text{TP+FP}}$ (S.1)

Sensitivity = $\frac{\text{TP}}{\text{TP+FN}}$ (S.2)

Specificity = $\frac{\text{TN}}{\text{TN+FP}}$ (S.3)

Accuracy = $\frac{\text{TP+TN}}{\text{TP+FP+FN+TN}}$ (S.4)

where: TP = True Positive, FP = False Positive, TN = True Negative, FN = False Negative

Confusion Matrix:

|  | Positive Predicted | Negative Predicted |
| --- | --- | --- |
| Positive Observed  (Active) | TP | FN |
| Negative Observed  (Inactive) | FP | TN |

**Mold2 descriptors for QSAR modeling.** We calculated 777 topological and physicochemical Mold2 descriptors to investigate the biological effect of the selected compounds on TNF. The QSAR model contains structural information that describes the relation between functional groups in each molecule, by also taking into account important physicochemical properties. To identify the most relevant descriptors for predicting TNF inhibition, three different feature selection techniques have been employed, namely the CFS subset evaluator **(I)**, the InfoGainAttribute evaluator **(II)**, and the Gain Ratio Feature evaluator **(III)**.

The definition and a brief description of the 27 Mold2 descriptors selected by the feature selection methods used in each methodology are presented in S2 Table; the corresponding feature selection method(s) applied on each descriptor are shown in the second column and additional information for specific descriptors is provided as notes.
