# Supplementary material for: Cheminformatics-aided discovery of small-molecule Protein-Protein Interaction (PPI) dual inhibitors of Tumor Necrosis Factor (TNF) and Receptor Activator of NF-κB Ligand (RANKL)
Source: PLoS Comput Biol. 2017 Apr 20;13(4):e1005372. doi: 10.1371/journal.pcbi.1005372 (PMC5398486; doi:10.1371/journal.pcbi.1005372)
Supplement: S2 Text — (DOCX) [file pcbi.1005372.s021.docx]

**Results**

**NMR Data**

^1^H NMR spectra were recorded on a Bruker 250 MHz instrument using *d6*-DMSO solvent.

**Structure elucidation**

T23:4,4'-dimethoxy-2,2'5,5'-tetraacetoxybiphenyl or according to IUPAC [4-acetyloxy-2-(2,5-diacetyloxy-4-methoxyphenyl)-5-methoxyphenyl] acetate

δ in ppm 2.07 5,5’ OCOCH_3_ (s,6H) , 2.28 2,2’ OCOCH_3_ (s,6H) 6.96 3,3’ (s,2H), 7.07 6,6’ (s,2H) (S3 Figure)

T8:N-[(4-chlorobenzoyl)amino]-N'-[4-[5-thiophen-2-yl-3-(trifluoromethyl)pyrazol-1-yl]phenyl]sulfonylpyrrolidine-1-carboximidamide

δ in ppm 1.86 4 (t, 4H), 3.47 3 (t, 4H), 7.09 7 (dd, 1H), 7.18 6 (dd, 1H), 7.27 5 (s, 1H), 7.53 1,2 (dd, 4H), 7.66 4 (dd , 4H), 7.91 1’,2’ (dd, 4H), 9.05 CONH, 10.45 CONHNH (S4 Figure)

According to the NMR data both compounds have a purity of more than 95%.

**MS data**

MS data confirm the identity of T8 and T23. As it can be seen in S5 Figure, the molecular weight of T8 was found to be 622.9 in accordance with the NMR structure elucidation. The molecular weight of T8 was found to be 447 (M+1) in accordance with the NMR structure elucidation. Peak at 464.1 corresponds to M+H_2_O (see S6 Figure). The MS data confirm the high purity of the compounds as no other peaks nearby the molecular weights of T8 and T23 are observed. Thus, MS data again are in accordance with those of NMR which show purity of the compounds higher than 95%.

**Determination of dissociation constant (K_d_) from fluorescence measurements**

The dissociation constant is an indicator of the binding strength between two molecules. For the reaction:

$$P+L\leftrightarrow PL$$

K_d_ is expressed by the equation:

$K_{d}=\frac{[P][L]}{[PL]}$ (S.5)

where [P] is the concentration of free Protein, [L] is the concentration of free Ligand and [PL] is the Ligand-bound-Protein.

Differences in fluorescence intensity between the protein/ligand complex and free protein (or free ligand) were analysed as previously described in [Papaneophytou *et al.* (**2013**) *ACS Med Chem Lett. 4:* 137-141] (Eq. S6) in order to determine the dissociation constant (K_d_) of the various protein-ligand systems.

$F_{obs}=F_{BG}+MF_{P_{F}}[P_{F}]+FR\cdot MF_{P_{F}}\cdot\frac{([L_{T}]+[P_{T}]+K_{d}])\pm\sqrt{([L_{T}]+[P_{T}]+K_{d}])^{2}-4[P_{T}][L_{T}]}}{2}$ (S.6)

In Eq. (S6) F_obs_ is the observed fluorescence intensity; F_BG_ is the fluorescence background signal; $\mathrm{MF}_{L_{F}}$and *P_F_* are the molar fluorescence and concentration of free protein, respectively; *FR* is the fluorescence ratio of bound protein (or bound ligand in the case of a fluorescence ligand); *L_T_* and *P_T_* are the total concentrations of ligand and protein, respectively. A detailed analysis of the development of the fluorescence ligand-binding assay is given in [Papaneophytou *et al.* (**2013**) *ACS Med Chem Lett. 4:* 137-141].
